# Single- Versus Multi-Fraction Stereotactic Body Radiotherapy for Primary Lung Cancer and Pulmonary Oligometastases: A Systematic Review and Meta-Analyses

**DOI:** 10.1016/j.jtocrr.2026.101005

**Published:** 2026-04-21

**Authors:** Ishaan Pillay, Harriet Gee, Eric Hau, Anselm Ong, Val Gebski

**Affiliations:** a Radiation Oncology Network, Western Sydney Local Health District, Sydney, New South Wales, Australia; b University of Sydney, Sydney, New South Wales, Australia; c Genome Integrity Unit, Children’s Medical Research Institute, Westmead, New South Wales, Australia; d Centre for Cancer Research, Westmead Institute of Medical Research, Westmead, New South Wales, Australia; e NHMRC Clinical Trials Centre, University of Sydney, Sydney, New South Wales, Australia

**Keywords:** Stereotactic ablative body radiotherapy, Primary lung cancer, Pulmonary oligometastases, Single fraction, Multi-fraction

## Abstract

Stereotactic body radiation therapy (SBRT), the standard of care for medically inoperable early-stage NSCLC, is increasingly used for pulmonary oligometastases. Optimal fractionation—single fraction (SF) versus multiple fractions (MF)—remains unclear, as existing randomized studies lack power to confirm SF noninferiority in local control. This review assessed 2-year local failure (LF) rates for SF versus MF SBRT. PubMed, Ovid Medline, and the Cochrane Library were searched. Titles and abstracts were reviewed followed by full-text review. Studies reporting LF outcomes for SF or MF SBRT in early-stage NSCLC or pulmonary metastases were included. Preferred Reporting Items for Systematic Reviews and Meta-Analyses and Cochrane Handbook for Systematic Reviews guidelines were followed. Pooled estimates were calculated using fixed- and random-effects models. Primary lung cancers treated with biologically effective doses (BED_10_) ≥100 Gy demonstrated similar 2-year LF rates with SF and MF SBRT. However, unclear T1/T2 classification may contribute to selection bias, as MF is often used for larger tumors. SF SBRT was associated with an 8.25% higher LF rate in cohorts treated with BED_10_ <100 Gy. Pulmonary metastases treated with SF demonstrated a 4% higher LF rate compared with MF, although substantial heterogeneity limited confidence in this finding. Limited data demonstrated the highest 2-year LF rates (25.6%) in colorectal oligometastases. Overall, SF SBRT appears to provide similar 2-year LF outcomes to MF in primary lung cancer when adequate BED is delivered, although cautious guideline application is warranted given the predominance of retrospective studies and limited data for larger or radioresistant tumors. MF may offer modest benefit for pulmonary metastases.

## Introduction

Stereotactic body radiation therapy (SBRT) is an established treatment option for patients with early stage lung cancer who are medically inoperable or who decline surgery.^1^^,^^2^ With preservation of pulmonary function and high efficacy, it is a preferable option for many patients. Unlike conventional radiotherapy, SBRT allows for delivery of high doses to the tumor with steep dose gradients, narrow margins, and thus reduced toxicity to the organs at risk.^3^ Rigorous debate, particularly in the development of clinical practice guidelines, continues regarding the use of multiple-fraction (MF) versus single-fraction (SF) treatment for both primary lung cancer and pulmonary oligometastases amenable to SBRT. SF SBRT provides health economic advantages, minimizes patient time and costs, while mitigating interruptions in systemic therapies.^4^ Indeed, a recent systematic review^5^ concluded that local control rates in early stage lung cancer were not compromised when patients were treated with SF SBRT. However, this conclusion was based on small noncomparative studies^6^ and a limited number of randomized (phase II) trials,^7^, ^8^, ^9^ with studies severely underpowered to evaluate noninferiority for these clinical outcomes. There have been no phase 3 trials. Treating physicians remain concerned regarding optimal fractionation regimens, particularly for larger tumors and radio-resistant histologies. To the best of our knowledge, there are no randomized studies powered to evaluate noninferiority of SF for local control as the primary outcome—and are unlikely to be performed in the future. Thus, we performed a thorough systematic review and meta-analysis investigating 2-year local failure (LF) rates for MF and SF SBRT stratified by radiation dose in both early stage lung cancer and pulmonary metastases, together with the subgroup of metastases from colorectal cancer (CRC). The numerical assessment of these results is critical to understanding the limitations in the data which underpin current guidelines.

## Methods

### Literature Search Strategy

This systematic review and meta-analysis was performed in accordance with the Cochrane Handbook for Systematic Reviews of Interventions^10^ and was reported according to the Preferred Reporting Items for Systematic Reviews and Meta-Analyses guidelines. Two independent reviewers searched three primary databases, including PubMed, Ovid Medline, and Cochrane Library. The relevant search terms included (single versus or single fraction or one fraction or one versus) and (SBRT or SABR or stereotactic body) and (lung cancer or "pulmonary oligometastases or lung tumours"). Titles and abstracts were then independently reviewed by the two reviewers. After, a full-text analysis was performed and studies meeting the inclusion criteria were incorporated in this paper. Articles published in English reporting outcomes for either SF or MF SBRT in the setting of early stage NSCLC or pulmonary metastases were included in this study. Comparison studies were also included. Exclusion criteria included abstracts, pediatric, and non-English studies. Similarly, studies involving patients treated for SCLC, proton therapy, and papers analyzing patient outcomes in the retreatment setting were excluded. Concurrent systemic therapy was not an exclusion criterion in this study. Studies in which local control was not either the primary or secondary end points were excluded from consideration. Studies published earlier than 2000 were excluded to ensure that staging and treatment were relevant to current guidelines. To increase the yield, references within included studies were also reviewed and incorporated if deemed to be relevant to this paper.

### Outcomes and Strategy for Data Analyses

The primary end point of this study was the assessment of LF rates at 2 years post-treatment. In instances where specific 2-year local control data were not explicitly provided, information was derived from published curves when available. Studies were initially categorized into primary, metastatic, and colorectal subgroups. Subsequently, based on the biologically effective dose (BED), each paper was further classified into BED_10_ more than or equal to or less than 100 Gy categories. Recognizing the inherent heterogeneity in fractionation regimens within individual papers, and in instances where subgroup analyses were absent, end point data were included in their respective categories only if a significant majority (>90%) received a BED_10_ more than or equal to 100 or less than 100 Gy. Papers containing local control data from various primary sites including colorectal metastases were encompassed within both the metastatic and colorectal subgroups. The Newcastle-Ottawa Scale^11^ and Cochrane risk-of-bias tool^12^ were used to assess the quality of evidence from the cohort studies and randomized controlled trials (RCTs) respectively demonstrated in Supplementary Tables A1 to A3.

### Statistical Considerations

The paucity of randomized trials specifically designed to evaluate local recurrence resulted in the systematic review being based on observational series comprising of either MF or SF modalities stratified by BED_10_ more than or equal to 100 or less than 100 Gy. Such studies are challenging due to a wide disparity of (1) monitoring local recurrences, (2) duration of patient follow-up, (3) reporting of result, and (4) study sample sizes. To account for such disparity, if no events were observed in 24 months, the event rate was estimated by 2/5N, where N is the number of subjects at 2 years or, if not stated, at the beginning of the study.^13^ Using the empirical estimate of zero suggests that there is no risk of recurrence which is clearly not clinically appropriate, particularly when the study sample size is small. If 2-year event rates were not given, they were extracted by digitizing the published survival curves using the digitizeit software (version 2.5.3).^14^ If 3- or 4-year rates were published, these were used to impute 2-year estimates using an exponential distribution. As local control rates are reported as either absolute rates (ignoring censoring), cumulative incidence (possibly adjusted for competing risks), or through Kaplan-Meier estimates, analyses were based on 2-year LF rates. Event rates that provided more than 1.5 but not 2 years were carried forward to 2 years. As the variances for studies having a small 2-year prevalence rate are low (regardless of the sample size), the inverse variance method in the pooling process unduly may give a much higher weight to these studies. This issue can be alleviated using a logit transformation of the prevalence rate and these inverse variance-weighted average to pool the individual studies.^15^ Pooled estimates and confidence intervals (CIs) are back transformed to a proportion.^16^ For studies reporting the LF rate on the number of treated lesions, this rate is assumed to apply for the total patient cohort being considered. Pooled estimates for fixed and random-effect models are reported. Studies with mixed treatment modalities or varying BED_10_ doses were included only if more than 90% of the patients received the same treatment modality or BED_10_ dose. Pooling was performed separately on the cohorts SF, BED_10_ less than 100 Gy; SF, BED_10_ more than or equal to 100 Gy; MF, BED_10_ less than 100 Gy; and MF, BED_10_ more than or equal to 100 Gy.

### Significant Heterogeneity Among Observational Studies

In observational studies, pooled 2-year LF estimates could be expected to exhibit high variability and heterogeneity, making clear interpretation of a priori statements of differences between treatment modalities/doses challenging. In these instances, traditional estimates such as CIs (which measure the precision of the treatment effect) lose interpretative value. The alternative is to calculate prediction intervals (PI), typically 95%, measuring the plausible range where, for future studies, the true treatment effects would be expected to lie.^17^^,^^18^ In the presence of heterogeneity, these intervals provide bounds on the spread of treatment effects in future studies. When there is little heterogeneity, confidence and prediction intervals would be numerically similar. The study protocol initially targeted a 5% noninferiority margin between MF and SF on the assumption that heterogeneity among studies within the respective cohorts would not be an issue. However, for low prevalence rates of LF (≤15%), this margin may be too optimistic. The presence of substantial heterogeneity between the studies precludes sensible interpretation of formal statistical comparison for noninferiority. All data pooling and estimation were performed using the Comprehensive Meta Analysis software, 2014, version 3.3, Biostat, Englewood, NJ. All statistical analysis was performed by VG.

## Results

**Figure 1:**
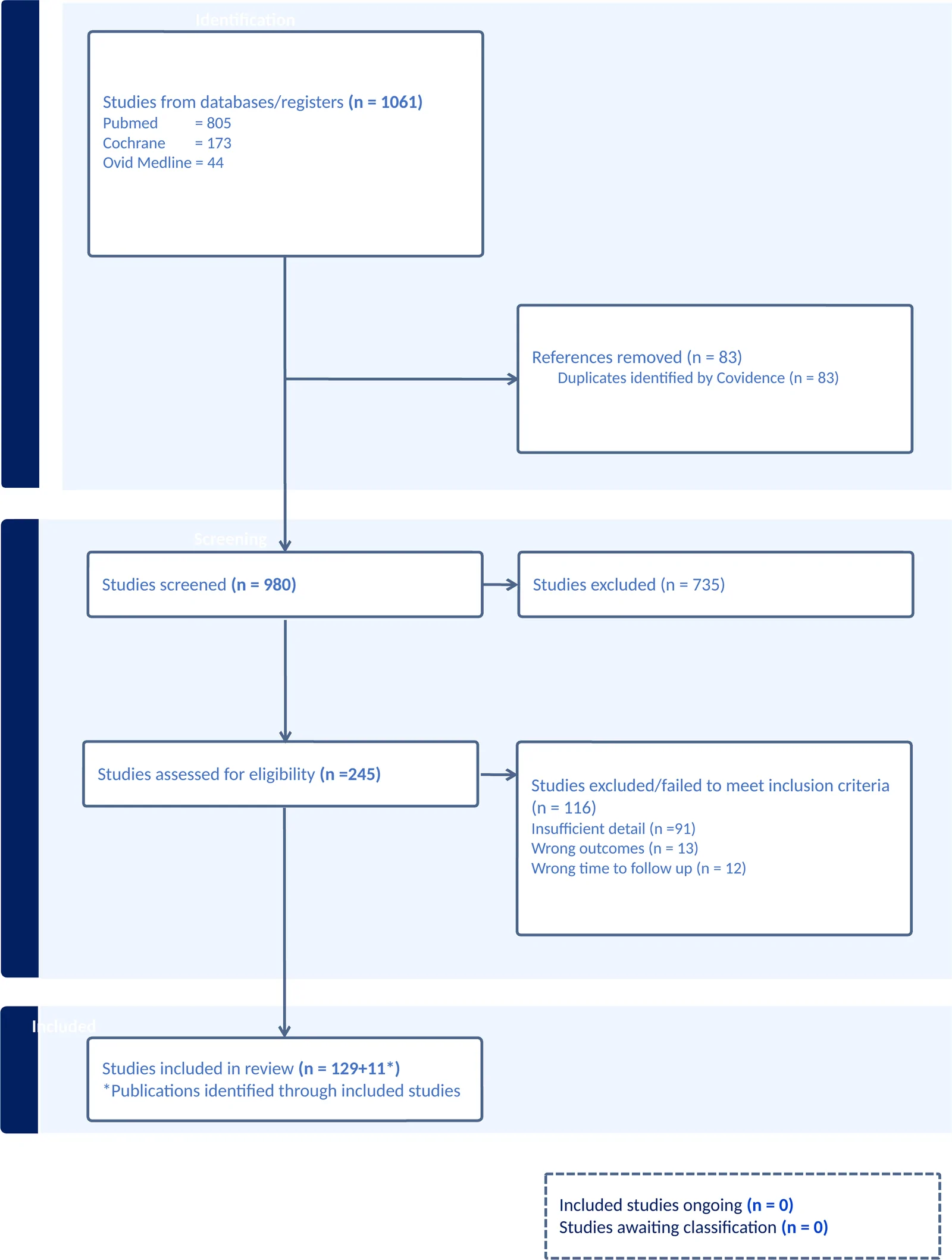
PRISMA flow diagram. PRISMA, Preferred Reporting Items for Systematic Reviews and Meta-Analyses.

A summary of study characteristics included in this meta-analysis is provided in Supplementary Tables 1 to 12, and study selection process is found in Figure 1.

### Primary Lung Cancer

The 2-year LF rates for primary lung cancer were identified in 10,850 patients from 120 reports. The pooled random-effect results were separated by treatment dose BED_10_ more than or equal to 100 Gy; less than 100 Gy and receiving either MF or SF SBRT are in Table 1 with corresponding forest plots in Supplementary Figure 1A to D. For treatments delivering BED10 more than or equal to 100 Gy, there is no evidence of differences in the 2-year LF rates between SF and MF schedules despite the MF studies exhibiting substantial heterogeneity, with a 2-year LF rate for a future study in this cohort having a 95% probability of being as high as 17%. The substantial heterogeneity from the 91 observational studies comprising 9119 patients is not unexpected, reflecting the diverse populations on which these studies are based.

**Table 1:** Pooled 2-Year Local Failure Results (Based on a Random Effect Model) for Patients With Primary Lung Cancer Receiving Multi and Single Fraction SBRT

Schedule		2-Y LF	95% CI		Patients	Reports		
	BED	Rate (%)	Lower	Upper	(N)	(n)	I^2^(%)	*p*(I^2^)
Multi	≥100 Gy	8.56	7.58	9.65	9119	91	61	<0.01
	95% prediction interval: 0%–17.5%							
Single		8.07	6.62	9.80	1285	13	<0.01	0.67
	95% prediction interval: 6.6%–9.8%							
Multi	<100 Gy	21.01	12.38	33.35	365	11	74	<0.01
	95% prediction interval: 9%–33%							
Single		29.26	19.48	41.43	81	5	<0.01	0.63
	95% prediction interval: 21%–37%							

BED, biologically effective dose; CI, confidence interval; LF, local failure; SBRT, Stereotactic body radiation therapy.

Pooled LF estimate for prescribed BED_10_ dose less than 100 Gy was 8% higher for SF albeit with substantial heterogeneity among the MF studies. Although the prediction intervals suggest that a further SF study would have higher LF rates than a MF study, definitive inferences about the potential detriment of SF cannot be drawn.

### Pulmonary Metastases From Any Primary

Pooled 2-year LF results from studies for patients with lung metastases from any primary histology for MF and SF separated for the BED_10_ doses are given in Table 2 with the study forest plots in Supplementary Figure 2A to D. The pooled LF rate for the MF BED_10_ more than or equal to 100 cohort is (numerically) lower than both SF BED_10_ more than or equal to 100 Gy and MF BED_10_ less than 100 Gy. The substantial heterogeneity for all three groups yields an uninformative predictive interval. The SF BED_10_ less than 100 Gy group demonstrates an unexpected low LF rate, the interpretation of which is clinically challenging. This cohort, however, comprises four studies and 188 patients which would not rule out substantial selection bias.

**Table 2:** Pooled 2-Year Local Failure Results (Based on a Random Effect Model) for Patients With Lung Cancer Metastases From Any Histologic Site Receiving Multi and Single Fraction SBRT

	Metastatic Lung Cancer							
Schedule		2-Y LF	95% CI		Patients	Reports		
	BED	Rate (%)	Lower	Upper	(N)	(n)	I^2^(%)	*p*(I^2^)
Multi	≥100 Gy	17.21	13.89	21.14	1698	32	68	< 0.01
	95% prediction interval: 0–100%							
Single		21.05	9.98	39.06	199	3	80	<0.01
	95% prediction interval: 0–89.6%							
Multi	<100 Gy	29.24	15.61	47.99	429	7	91	<0.01
	95% prediction interval: 0–100%							
Single		15.49	10.94	21.47	188	4	<0.01	0.69
	95% prediction interval: 10–21%							

BED, biologically effective dose; CI, confidence interval; LF, local failure; SBRT, Stereotactic body radiation therapy.

### Pulmonary Metastases From CRC

**Figure 2:**
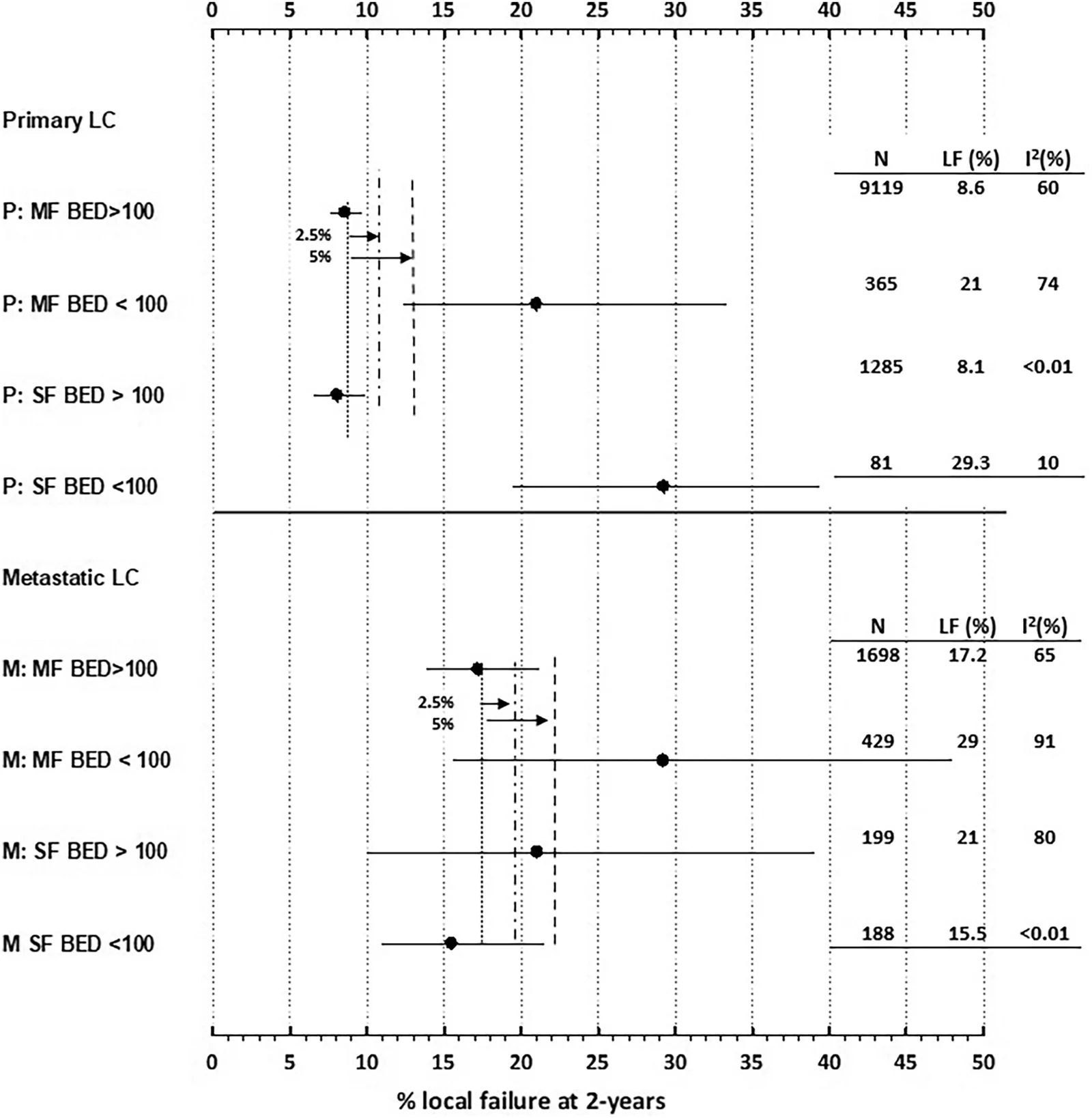
Summary: primary lung cancer and pulmonary metastases from any primary for BED more than or equal to 100 Gy and BED less than 100 Gy. 2.5% and 5% margins (reference: multi-fraction, BED > 100 Gy), N = total number of patients. BED, biologically effective dose; LC, lung cancer; LF, local failure; MF, multi-fraction; SF, single fraction.

**Figure 3:**
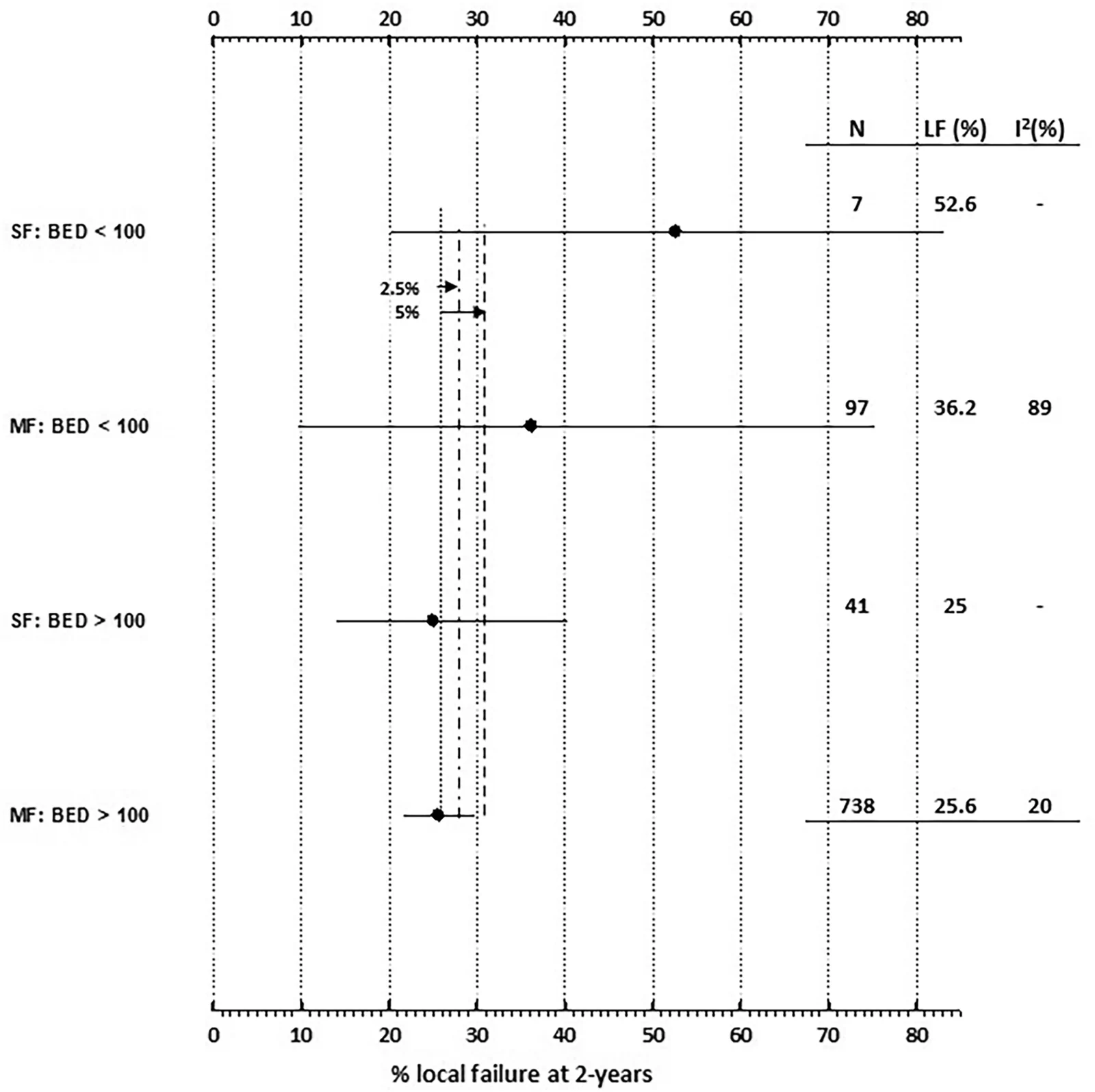
Summary: pulmonary metastases from CRC for BED_10_ ≥ more than or equal to 100 Gy and BED_10_ less than 100 Gy. 2.5% and 5% margins (reference: multi-fraction, BED_10_ > 100 Gy), N = total number of patients. BED, biologically effective dose; CRC, colorectal cancer; LF, local failure; MF, multi-fraction; SF, single fraction.

Table 3 gives pooled results for 2-year LF, with corresponding forest plots in Supplementary Figure 3A to D. The LF in studies having doses BED_10_ more than or equal to 100 Gy demonstrates substantially lower LF rate than those with BED_10_ less than 100 Gy. The inclusion of only one study in the SF cohort precludes definitive conclusions regarding LF outcomes. Results for primary lung cancer plus pulmonary metastatic disease from any primary histology and from colorectal primary are illustrated in Figure 2 and Figure 3, respectively.

**Table 3:** Pooled 2-Year Local Failure Results (Based on a Random Effect Model) for Patients With Lung Cancer Metastases From Colorectal Primary Receiving Multi and Single Fraction SBRT

	Metastatic Lung Cancer from colorectal primary							
Schedule		2-Y LF	95% CI		Patients	Reports		
	BED	Rate (%)	Lower	Upper	(N)	(n)	I^2^(%)	*p*(I^2^)
Multi	≥100 Gy	25.59	21.80	29.79	738	17	20	0.22
	95% prediction interval: 0–66%							
Single		25.02	14.13	40.35	41	1		
Multi	<100 Gy	36.19	9.60	75.17	97	3	89	<0.01
	95% prediction interval: 0–100%							
Single		52.60	20.11	83.03	7	1		

BED, biologically effective dose; CI, confidence interval; LF, local failure; SBRT, Stereotactic body radiation therapy.

BED_10_ more than or equal to 100 Gy is used as a reference group with 2.5% and 5% boundaries highlighting what would be considered acceptable departure from this reference.

### SBRT for Pulmonary Metastases From Any Primary

**Figure 4:**
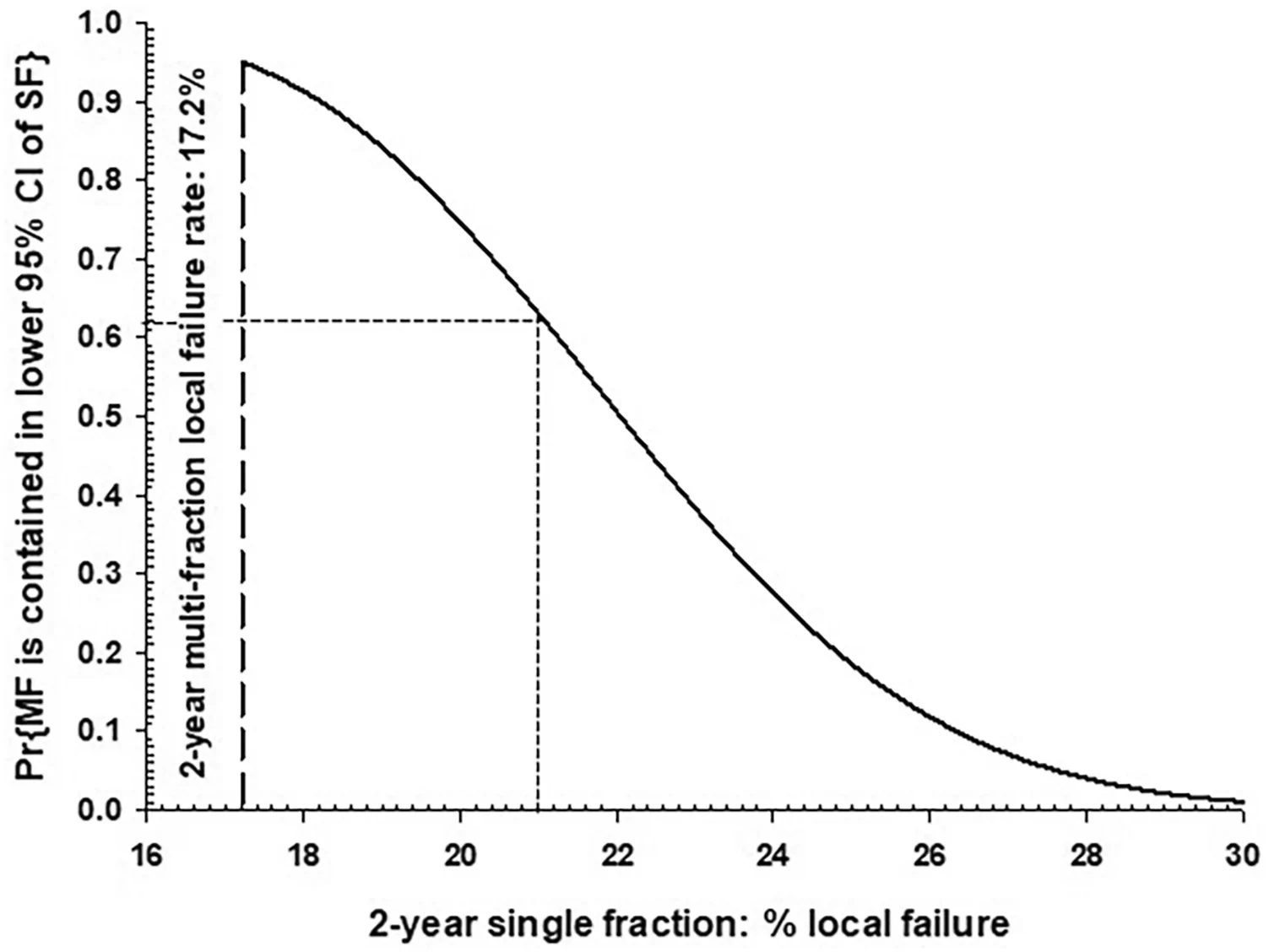
The 2-year local control confidence distribution for metastatic disease from any primary: BED_10_ >100 Gy single fraction compared with multi-fraction. Single fraction: LF 21%, N = 199; multi-fraction: LF 17%, N = 1698. Pr (21% is consistent with 17%) < 63%. BED, biologically effective dose; CI, confidence interval; LF, local failure; MF, multi-fraction; SF, single fraction.

Metastatic lesions arose from multiple subsites with the most common being CRC and head, neck, and lung primaries. Issues regarding interpretation of pooled estimates from a random-effect adjustment are also affected when groups being compared are based on substantially different sample sizes. The BED_10_ more than or equal to 100 Gy LF estimates for MF schedule in Table 2 are based on 1698 cases (32 reports), eight times the size of the SF cohort of 188 cases (four reports) with the estimates having overlapping CIs. Interpretation regarding the issue of disproportionate sample sizes can in part be addressed by considering the confidence distributions or fiducial probabilities.^19^ To illustrate, setting aside the issue of heterogeneity, the pooled 2-year LF rate for the MF group receiving BED_10_ ≥100 Gy was 17.2% (Fig. 2), representing the standard-of-care cohort treated with multifraction radiation therapy. For different values of 2-year LF rates in the SF BED_10_ >100 Gy group, the probabilities that the SF rate differs from 17.2% can then be calculated. The 1698 cases in Table 2 could be considered sufficiently large to approximate the underlying population value. Treating 17.2% as fixed, different 2-year LF rates in the SF group can be obtained to generate confidence curve. This is illustrated in Figure 4.

This curve provides the probability that the lower 95% CI for a fixed 2-year LF rate in the SF group is consistent with the MF rate of 17.2%. For our studies, the probability that the observed SF estimate of 21% is consistent with the MF estimate of 17.2% is less than 63%. Considering the extensive heterogeneity between the studies, caution should be exercised when interpreting this probability.

## Discussion

Although comprehensive evidence for noninferiority of SF to MF treatment of patients with lung cancer is nonexistent, there is nevertheless growing support for SF treatment to be included in practice guidelines. Our review has highlighted major limitations among the published reports, in particular (1) the lack of sufficiently powered RCTs addressing noninferiority of 2-year LF, (2) studies analyzing the number of lesions as if these were individual patients (ignoring within patient correlation), (3) lack of sufficient long-term follow-up to provide reliable 2-year LF estimates, and (4) use of suboptimal statistical methods (e.g., ignoring competing risks) to calculate the 2-year LF. Furthermore, the lack of clarity regarding the classification of cases as either T1 or T2 limits our capacity to conclusively rule out selection bias. This is due to the frequent application of MF for larger tumors. Despite these shortcomings, we attempted to synthesize the available information to gain insight into whether SF SBRT was a clinically acceptable treatment modality.

### SBRT for Primary Lung Cancer

For primary lung cancer, there was little separation in 2-year LF estimates between the two modalities for patients treated with “adequate” (BED_10_ ≥ 100 Gy) dose. Lower radiation doses (BED_10_ < 100 Gy) demonstrated an 8.25% increase in LF for SF while acknowledging that for the MF cohort in both dose groups; a substantial degree of heterogeneity between the studies was observed reflecting differing patient risk profiles being selected for treatment. More explicitly, as all but three studies were predominantly retrospective or cohort designs, it is unknown whether there was strong clinical selection bias in the MF group of larger tumors or more radio-resistant histologies (e.g., some squamous cell carcinomas), as these data which could influence local control were not linked to dose or outcome. These results are consistent with claims of comparable efficacy between SF and MF SBRT with respect to local control for primary lung cancer.^6^^,^^8^^,^^20^^,^^21^

### SBRT for Pulmonary Metastases From Any Primary

In studies with BED_10_ more than or equal to 100 Gy for pulmonary metastases from any primary, there was a 4% higher 2-year LF rate for SF SBRT. In the presence of substantial heterogeneity, this result should be interpreted with caution. Due to there being an 8.5-fold difference between the group sample sizes, the confidence distribution gives a probability of less than 63% that the SF estimate may be considered consistent with the MF. Studies of low radiation doses give counterintuitive results; the LF estimate for the SF dose being 53% lower than relative to the MF regimen. In this setting, the LF estimate for BED_10_ less than 100 Gy SF being lower than both MF and SF estimates for BED_10_ more than or equal to 100 Gy regimens strongly suggests patient selection bias (higher proportion of T1 tumors in the low-dose SF group) as a possible explanation. A conclusion that BED_10_ less than 100 Gy SF dose is noninferior to MF or BED_10_ more than or equal to 100 Gy SF regimens is not warranted without further evidence from well-designed studies.

### SBRT for Pulmonary Metastases From CRC

CRC constitutes the second most common cause of pulmonary metastases with close to 20% of patients presenting with metastatic disease at the time of diagnoses.^22^ The radio-resistant nature of CRC often leads to poorer LC rates with a recent study by Verma et al.^23^ demonstrating higher rates of LF despite BED_10_ doses more than or equal to 100 Gy. Several factors including down-regulation of miR-423-5p and expression of the apoptosis protein surviving have been implicated in the mechanism of radioresistance.^24^^,^^25^ In this review, a higher incidence of LF for CRC at 2 years (25.6%) was found for MF BED_10_ more than or equal to 100 Gy when compared with primary lung cancer (8.6%) and pulmonary metastases from any primary (17.2%). The availability of only individual papers for comparison in the SF cohort impeded our ability to conduct a meaningful analysis between SF and MF. Paucity of data for pulmonary metastatic disease secondary to CRC failed to provide any meaningful interpretation between the various dose subgroups. There are insufficient data to conclude that, for lung metastases in CRC, a SF is as effective as MF. Compared with primary lung cancer and pulmonary metastases, the higher incidence of LF rate at 2 years (25.6%) for MF BED_10_ more than or equal to 100 Gy in this cohort supports results found in previous studies.^23^^,^^26^

Higher LF rates observed with BED_10_ less than 100 Gy across all groups (apart from metastatic disease of any primary) are unsurprising and consistent with existing literature.^23^^,^^27^

This study was limited by the extent of heterogeneity observed within the dose groups and different treatment modalities. The significance of concurrent systemic therapies on LF rates is difficult to determine. Insufficient information for T1 and T2 tumors and for different histologic subtypes of primary lung cancer prevented comparisons of LF rates between these categories. We note that the recent iSABR trial revealed that higher BED and MF treatments for larger tumors provide local control comparable to SF therapy for smaller tumors.^28^ Apart from these limitations, the requirement for 2-year LF imputation (from either 3-y LF rates or zero failures) and the need to estimate rates from the published LF curves add to the requirement for cautious interpretation of the results. Despite these limitations, we have provided a comprehensive synthesis of the available evidence to evaluate differences between MF and SF SBRT, with outcomes stratified according to delivered radiation dose using a BED_10_ threshold of more than or equal to 100 Gy versus less than 100 Gy. Although at this stage it would be difficult to initiate an RCT, the assumption of SF noninferiority (apart from primary lung cancer BED_10_ ≥ 100 Gy) is not warranted.

## Conclusion

Based on the currently available evidence, there is no clear signal that SF SBRT delivering BED_10_ more than or equal to 100 Gy results in inferior 2-year LF rates compared with MF regimens in lung cancer. However, interpretation should be cautious, as most included studies were retrospective and treatment selection may have been influenced by clinical or logistical factors, including performance status and tumor characteristics, introducing potential selection bias.

Accordingly, clinical decision-making should remain individualized, particularly for larger tumors or potentially radio-resistant histologies.

For pulmonary metastases treated with BED10 more than or equal to 100 Gy, MF schedules appear to demonstrate a modest advantage than SF in some series; however, these findings are limited by substantial heterogeneity and small patient numbers. For colorectal metastases specifically, available data remain insufficient to support definitive conclusions regarding optimal fractionation, although outcomes overall suggest relative radioresistance.

Across studies, BED less than 100 Gy SF regimens were generally associated with poorer local control, supporting the importance of adequate biological dose irrespective of fractionation approach.

## CRediT Authorship Contribution Statement

**Ishaan Pillay:** Writing - original draft, Methodology, Investigation, Data curation.

**Anselm Ong:** Writing - original draft, Investigation.

**Eric Hau**: Conceptualization, Supervision, Writing - review & editing, Project administration.

**Harriet Gee:** Conceptualization, Supervision, Writing - review & editing, Project administration.

**Val Gebski:** Data curation, Investigation, Formal analysis.

## Supplementary Data

Note: To access the supplementary material accompanying this article, visit the online version of the *JTO Clinical and Research Reports* at www.jtocrr.org and at https://doi.org/10.1016/j.jtocrr.2026.101005.

## Disclosure

Dr. Hau has received honoraria from Novartis and honoraria and research funding from AstraZeneca. These activities had no impact on the design or findings of the submitted manuscript. The remaining authors declare no conflict of interest.
