# Supplementary material for: Single- Versus Multi-Fraction Stereotactic Body Radiotherapy for Primary Lung Cancer and Pulmonary Oligometastases: A Systematic Review and Meta-Analyses
Source: JTO Clin Res Rep. 2026 Apr 21;7(9):101005. doi: 10.1016/j.jtocrr.2026.101005 (PMC13508619; doi:10.1016/j.jtocrr.2026.101005)
Supplement: Supplementary file 1 [file mmc1.docx]

**SUPPLEMENTARY MATERIAL:**

**Published reports used in this meta-analysis:**

eTable 1: **Primary lung Cancer, BED ≥ 100Gy multi fraction SBRT**

| *Study* | *No. of patients* | *Male %* | *Dose/Median BED* | *2-yr Local failure %* |
| --- | --- | --- | --- | --- |
| Abdalmassih 2020^1^ | 158 | 40 | 60Gy/8fraction (n=32); 48Gy/4fraction (n=121) | 13 |
| Abe 2021^2^ | 26 | 85 | 54Gy/3fraction | 1.54 |
| Abreu 2018^3^ | 54 | 76 | 54Gy/3fraction(median) | 10.9 |
| Alongi 2021^4^ | 29 | 79 | 70Gy/10fraction | 3.8 |
| Aoki 2018^5^ | 35 | 71 | 56Gy/7fraction | 4 |
| Arnett 2019^6^ | 103 | 49 | Median BED_10_ 100Gy | 5 |
| Baumann 2009^7^ | 57 | 46 | 45Gy/3fraction | 5.4 |
| Bezjak 2019^8^ | 71 | 39 | 11.5-12Gy/5fraction | 11.3 |
| Bhandari 2016^9^ | 55 | 80 | Median BED_10_ 100Gy | 9 |
| Bral 2011^10^ | 40 | 83 | 60Gy/3-4fraction | 16 |
| Chang 2012^11^ | 130 | 52 | 50Gy/4fraction | 1.5 |
| Chang 2021^12^ | 80 | 55 | 50-54Gy/3-4fraction | 3.7 |
| Chaudari 2015^13^ | 68 | 43 | 50Gy/4-5fraction | 10 |
| Chiang 2016^14^ | 57 | 28 | 48-52Gy/4-5fraction | 4.4 |
| Choi 2023^15^ | 38 | NA | 50-56Gy/4-5fraction | 6.7 |
| Cummings 2018^16^ | 98 | 45 | Median dose of 50Gy/5fraction | 10 |
| Doupnik 2022^17^ | 44 | NA | 48Gy/4fraction or 60Gy/8fraction | 6 |
| Dubaere 2019^18^ | 189 | 66 | 48Gy/4fraction | 15 |
| Factor 2014^19^ | 74 | 46 | Median dose of 48Gy/4fraction | 13 |
| Fakiris 2008^20^ | 70 | NA | 60-66Gy/3fraction | 8.1 |
| Fischer-Valuck 2015^21^ | 88 | 47 | 48-60Gy/4-5fraction | 7 |
| Grills 2010^22^ | 58 | 40 | 48Gy/4fraction, 60Gy/5fraction | 4 |
| Haasbeek 2010^23^ | 193 | 68 | 20Gy/3fraction, 12Gy/5fraction, 7.5Gy/8fraction | 5.2 |
| Hamamoto 2009^24^ | 52 | 69 | 48Gy/4fraction | 12 |
| Hayashi 2022^25^ | 153 | 67 | 52Gy/4fraction | 8.1 |
| Heal 2015^26^ | 100 | 53 | Median BED_10_ 151Gy | 6 |
| Hobbs 2017^27^ | 282 | 46 | 48Gy/4fraction, 54Gy/3fraction, 50Gy/5fraction ***** | 5 |
| Hoyer 2006^28^ | 40 | 45 | 15Gy/3fraction | 15 |
| Huang 2023^29^ | 74 | 53 | 50Gy/5fraction | 7.16 |
| Jeppesen 2013^30^ | 100 | 45 | 66Gy/3fraction,45Gy/3fraction | 19.1 |
| Karasawa 2018^31^ | 56 | 70 | 48Gy/4fraction | 16.6 |
| Kim 2013^32^ | 16 | 75 | 48Gy/4fraction, 55Gy/5fraction | 9 |
| Koba 2020^33^ | 95 | 71 | 50Gy/4fraction, 50Gy/5fraction **^*^** | 22 |
| Koto 2007^34^ | 31 | 80 | 45Gy/3fraction, 60Gy/8fraction | 28.2 |
| Kowalchuk 2021^35^ | 98 | NA | Median dose of 45Gy/4fraction | 23.1 |
| Lagerwaard 2008^36^ | 206 | 56 | 20Gy/3fraction, 12Gy/5fraction, 7.5Gy/8fraction | 7 |
| Lee 2018^37^ | 169 | 78 | 60Gy/4fraction, 48Gy/4fraction **^*^** | 11.7 |
| Lenglet 2019^38^ | 137 | 45 | Median BED_10_ 100Gy | 18.6 |
| Li 2014^39^ | 82 | 53 | 70Gy/10fraction | 3.7 |
| Lischalk 2016^40^ | 61 | 33 | 50Gy/5fraction, 45Gy/3fraction, 54Gy/3fraction **^*^** | 1.9 |
| Louie 2014^41^ | 9 | 51 | 20Gy/3fraction, 12Gy/5fraction **^*^** | 1.1 |
| Lucas 2014^42^ | 81 | 48 | Median dose of 54Gy/3fraction | 7.1 |
| Ma 2018^43^ | 94 | 50 | Median 60Gy/3fraction | 13.8 |
| Mancini 2016^44^ | 251 | 55 | Median BED_10_ 151.2Gy | 12.8 |
| Matsuo 2011^45^ | 101 | 73 | 48Gy/4fraction | 8.3 |
| Meng 2019^46^ | 80 | 70 | Median BED_10_ 102.6Gy | 4 |
| Mihai 2019^47^ | 200 | 54 | 60Gy/8fraction, 60Gy/5fraction **^*^** | 9.6 |
| Mix 2019^48^ | 297 | 52 | 48Gy/4fraction, 54Gy/3fraction, 50Gy/5fraction **^*^** | 5.7 |
| Miyakawa 2013^49^ | 91 | 71 | 48 - 52Gy/4fraction | 11.4 |
| Miyakawa 2017^50^ | 71 | 72 | 48 - 52Gy/4fraction | 7.5 |
| Nagata 2005^51^ | 45 | 73 | 48Gy/4fraction | 2 |
| Nagata 2015^52^ | 164 | 74 | 48Gy/4fraction | 8.6 |
| Nath 2011^53^ | 48 | 62 | Median 48Gy/4fraction | 5 |
| Ng 2008^54^ | 20 | 80 | 54Gy/3fraction, 45Gy/3fraction **^*^** | 5.3 |
| Nittala 2021^55^ | 114 | 63 | 50-55Gy/5fraction | 16.7 |
| Nyman 2016^56^ | 49 | 45 | 66Gy/3fraction | 9.2 |
| Olsen 2011^57^ | 122 | 60 | 18Gy/3fraction, 10Gy/5fraction ***** | 8 |
| Onimaru 2008^58^ | 41 | 68 | 48Gy/4fraction **^*^** | 27 |
| Onishi 2011^59^ | 87 | 72 | Median BED_10_ 116Gy | 4.7 |
| Patel 2020^60^ | 152 | 55 | 54Gy/3fraction, 48-52.5Gy/4-5fraction | 5 |
| Raman 2018^61^ | 206 | 47 | 60Gy/8fraction. 48Gy/4fraction **^*^** | 3.3 |
| Ricardi 2010^62^ | 62 | 84 | 45Gy/3fraction | 8.3 |
| Ricardi 2014^63^ | 196 | 75 | 48Gy/4fraction, 45Gy/3fraction, 50Gy/5fraction **^*^** | 9.1 |
| Roman 2020^64^ | 60 | NA | 54Gy/3fraction, 50Gy/5fraction **^*^** | 9.7 |
| Ryuno 2022^65^ | 96 | 64 | 54Gy/3fraction, 60Gy/3fraction | 3.6 |
| Scotti 2019^66^ | 93 | NA | NA (BED_10_ >100 for all patients) | 9.6 |
| Shintani 2018^67^ | 216 | 77 | 48Gy/4fraction | 13.2 |
| Shirata 2012^68^ | 80 | 80 | 48Gy/4fraction, 60Gy/8fraction **^*^** | 7.4 |
| Singh 2019^69^ | 49 | 53 | 60Gy/3fraction | 2.9 |
| Song 2009^70^ | 32 | 81 | 48Gy/4fraction **^*^** | 16.1 |
| Song 2023^71^ | 40 | 78 | 60Gy/10fraction, 56gy/7fraction **^*^** | 7.9 |
| Stephans 2018^72^ | 600 | 47 | 60Gy/3fraction, 50Gy/5fraction ***** | 13.1 |
| Sun 2017^73^ | 65 | 49 | 50Gy/4fraction | 3.0 |
| Takeda 2009^74^ | 63 | 63 | 50Gy/5fraction | 5.8 |
| Takeda 2011^75^ | 183 | 70 | 50Gy/5fraction | 7 |
| Taremi 2012^76^ | 108 | 49 | 48Gy/4fraction, 60Gy/3fraction **^*^** | 8.8 |
| Timmerman 2010^77^ | 55 | 38 | 54Gy/3fraction | 1.6 |
| Timmerman 2018^78^ | 26 | 58 | 54Gy/3fraction | 4 |
| Trakul 2012^79^ | 31 | 45 | Median BED of 112.5Gy | 9.1 |
| Turzer 2011^80^ | 36 | 36 | 45Gy/3fraction | 1.1 |
| Van Der Voort 2009^81^ | 70 | NA | 60Gy/3fraction **^*^** | 6.8 |
| Verstegen 2011^82^ | 591 | 60 | 60Gy/3fraction, 60Gy/5fraction, 60Gy/8fraction | 5.5 |
| Videtic 2019^83^ | 45 | 49 | 48Gy/4fraction | 2.2 |
| Wang 2018^84^ | 46 | 39 | 48Gy/4fraction* | 2.8 |
| Watanabe 2021^85^ | 64 | 70 | 48Gy/4fraction **^*^** | 5.5 |
| Wegner 2018^86^ | 196 | 53 | Median dose 48Gy/4fraction | 6 |
| Xia 2006^87^ | 43 | 72 | 70Gy/10fraction | 3.3 |
| Yamashita 2014^88^ | 51 | 72 | 50-55Gy/4fraction * | 8 |
| Yang 2020^89^ | 21 | 57 | 60Gy/8fraction | 7.1 |
| Yaprak 2019^90^ | 52 | 46 | Median dose of 45Gy/3fraction | 15.4 |
| Yoshitake 2015^91^ | 88 | 55 | 48Gy/4fraction | 4.6 |

*Most commonly used dose prescription/s in the study; NA: Not provided

etable 2: **Primary lung Cancer, BED < 100Gy multi fraction SBRT**

| *Study* | *No. of patients* | *Male %* | *Dose/Median BED* | *2-yr Local failure %* |
| --- | --- | --- | --- | --- |
| Ahmed 2018^92^ | 18 | 33. | Median BED 72Gy | 13 |
| Kundu 2013^93^ | 8 | 88 | 48Gy/6fraction, 48GY/8fraction | 5 |
| Lodeweges 2021^94^ | 72 | 63 | 60Gy/12fraction | 15 |
| Nestle 2020^95^ | 56 | NA | 3x12.5Gy, 5x7Gy* | 7.3 |
| Olsen 2011^57^ | 8 | NA | 45Gy/5fraction | 50 |
| Onimaru 2008^58^ | 13 | NA | 40Gy/4fraction | 56.8 |
| Schanne 2015^96^ | 90 | 69 | Median BED of 72Gy | 36 |
| Shirata 2012^68^ | 7 | NA | 60Gy/15fraction | 43 |
| Testolin 2015^97^ | 12 | 67 | 40Gy/4fraction, 30Gy/3fraction * | 35.5 |
| Wulf 2004^98^ | 20 | 70 | 37.5Gy/3fraction, 36Gy/3fraction * | 5 |
| Zimmerman 2005^99^ | 68 | NA | 37.5Gy(mean dose)/3-5fraction | 12 |

*Most commonly used dose prescription/s in the study; NA: Not provided

eTable 3: **Primary Lung Cancer, BED ≥ 100Gy single fraction SBRT**

| *Study* | *No. of patients* | *Male %* | *Dose/Median BED* | *2-yr Local failure %* |
| --- | --- | --- | --- | --- |
| Alongi 2021^4^ | 44 | 66 | 30Gy/1fraction | 12.2 |
| Cummings 2018^16^ | 65 | 4 | 30Gy/1fraction | 12 |
| Fritz 2006^100^ | 33 | NA | 30Gy/1fraction | 1.2 |
| Fritz 2008^101^ | 40 | 80 | 30Gy/1fraction | 4.9 |
| Huang 2023^29^ | 191 | 44 | 27Gy/1fraction | 6.4 |
| Iovoli 2023^102^ | 263 | 43 | 27-30Gy/1fraction | 8 |
| Ma 2018^43^ | 65 | 46 | 30Gy/1fraction | 12.2 |
| Manyam 2019^103^ | 139 | 47 | 30-34Gy/1fraction | 7.3 |
| Nicosia 2019^104^ | 44 | 66 | 30Gy/1fraction | 9.2 |
| Singh 2019^69^ | 49 | 45 | 30Gy/1fraction | 5.1 |
| Stephans 2018^72^ | 84 | 56 | 30-34Gy/1fraction | 13.1 |
| Videtic 2019^83^ | 39 | 41 | 34Gy/1fraction | 2.6 |
| Videtic 2021^105^ | 229 | 46 | 30Gy/1fraction, 34Gy/1fraction | 7.3 |

eTable 4: **Primary Lung Cancer, BED < 100Gy single fraction SBRT**

| *Study* | *No. of patients* | *Male %* | *Dose/Median BED* | *2-yr Local failure %* |
| --- | --- | --- | --- | --- |
| Hof 2003^106^ | 10 | 100 | 19-26Gy/1fraction | 28.9 |
| Hof 2007^107^ | 10 | 88 | 26Gy/1fraction, 28Gy/1fraction* | 50 |
| Le 2006^108^ | 32 | NA | 25Gy/1fraction* | 31 |
| Testolin 2015^97^ | 5 | NA | 25Gy/1fraction | 40 |
| Trakul 2012^79^ | 36 | NA | Median dose 25Gy/1fraction | 17.8 |

*Most commonly used dose prescription/s in the study; NA: Not provided

eTable 5: **Pulmonary metastatic disease from any primary, BED ≥ 100Gy multi fraction SBRT**

| *Study* | *No. of patients* | *Male %* | *Dose/Median BED* | *2-yr Local failure %* |
| --- | --- | --- | --- | --- |
| Baschnagel 2013^109^ | 32 | NA | Median 60Gy/4fraction | 8 |
| Berkovic 2020^110^ | 104 | 53 | 60Gy/3fraction | 20 |
| Binkley 2015^111^ | 23 | NA | 50Gy/4fraction* | 7.9 |
| Dohopolski 2018^112^ | 105 | 51 | 60Gy/3fraction, 48Gy/4fraction* | 6 |
| Francheschini 2017^113^ | 200 | 62 | 48Gy/4fraction, 60Gy/8fraction * | 15.1 |
| Garcia-Cabezas 2015^114^ | 44 | 86 | 60Gy/5fraction, 60G/8fraction * | 13.3 |
| Hamamoto 2010^24^ | 10 | 30 | 48Gy/4fraction | 75 |
| Helou 2017^115^ | 120 | 48 | 48-52Gy/4-5fraction | 15.2 |
| Jingu 2017^116^ | 22 | 65 | Median BED 105Gy | 4.9 |
| Jung 2015^117^ | 50 | NA­­ | 48Gy/4fraction, 60Gy/4fraction * | 27.1 |
| Kim 2009^118^ | 13 | 46 | Median 45Gy/3fraction | 47.3 |
| Kinj 2017^119^ | 53 | 34 | Median BED 180Gy | 21.8 |
| Kobayashi 2020^120^ | 20 | 65 | 54-60Gy/3fraction | 34.2 |
| Lee 2018^121^ | 21 | 57 | 60Gy/3fraction or 48Gy/4fraction | 24.8 |
| Li 2019^122^ | 35 | 81 | 60Gy/10fraction, 50GY/10fraction* | 3.7 |
| Louie 2014^41^ | 11 | N/A | 54Gy/3fraction, 55Gy/5fraction* | 3.6 |
| Navarria 2014^123^ | 76 | 70 | 48Gy/12fraction * | 11 |
| Nicosia 2020^124^ | 38 | NA | 50-55Gy/5fraction, 70Gy/10fraction * | 20 |
| Norihisa 2008^125^ | 34 | 65 | 48-60Gy/4fraction | 10 |
| Oh 2012^126^ | 57 | 86 | 60Gy/5fraction, 60Gy/4fraction * | 2.5 |
| Ricco 2017^127^ | 178 | NA | Median BED 100Gy | 26.3 |
| Roman 2020^64^ | 31 | NA | 54Gy/3fraction, 50Gy/5fraction * | 13.5 |
| Rusthoven 2009^128^ | 38 | NA | 48-60Gy/3fraction | 4 |
| Siva 2015^129^ | 24 | 58 | 48Gy/4fraction, 50Gy/5fraction | 8 |
| Siva Safron II 2021^130^ | 45 | 67 | 48Gy/4fraction | 20 |
| Takeda 2011^75^ | 34 | 79 | 50Gy/5fraction | 18 |
| Takeda 2016^131^ | 9 | 71 | 50-60Gy/5fraction | 4.4 |
| Thibault 2014^132^ | 53 | NA | 48-60Gy/4-5fraction | 15.9 |
| Trakul 2012^79^ | 4 | NA | 50Gy/4fraction* | 8 |
| Wang 2018^84^ | 15 | 67 | 60Gy/5fraction* | 22.2 |
| Yamashita 2014^88^ | 16 | NA | 50-55GY/4fraction* | 25 |
| Yamamoto 2020^133^ | 183 | 61 | NA | 29.6 |

*Most commonly used dose prescription/s in the study; NA: Not provided

eTable 6: **Pulmonary metastatic disease from any primary, BED < 100Gy multi fraction SBRT**

| *Study* | *No. of patients* | *Male %* | *Dose/Median BED* | *2-yr Local failure %* |
| --- | --- | --- | --- | --- |
| Chai 2020^137^ | 84 | 62 | 60Gy/15fraction, 60Gy/10fraction* | 16.5 |
| Li 2019^122^ | 18 | NA | 60Gy/10fraction,50Gy/10fraction | 19.4 |
| Lischalk 2016^138^ | 20 | 50 | 35-40Gy/5fraction | 42.6 |
| Nestle 2020^95^ | 44 | NA | 3x12.5Gy, 35Gy/5fraction* | 9.2 |
| Okunieff 2006^139^ | 49 | 45 | 50Gy/10fraction | 17 |
| Qiu 2018^140^ | 65 | 57 | 50Gy/10fraction* | 69.1 |
| Ricco 2017^127^ | 149 | NA | NA | 46.9 |

*Most commonly used dose prescription/s in the study; NA: Not provided

eTable 7: **Pulmonary metastatic disease from any primary, BED ≥ 100Gy single fraction SBRT**

| *Study* | *No. of patients* | *Male %* | *Dose/Median BED* | *2-yr Local failure %* |
| --- | --- | --- | --- | --- |
| Fritz 2006^100^ | 25 | NA | 30Gy/1fraction | 17 |
| Osti 2018^136^ | 129 | 60 | 30Gy/1fraction | 13.8 |
| Siva 2021^130^ | 45 | 62 | 28Gy/1fraction | 32.7 |

NA: Not provided

eTable 8**: Pulmonary oligometastatic disease from any primary BED < 100Gy single fraction SBRT**

| *Study* | *No. of patients* | *Male %* | *Dose/Median BED* | *2-yr Local failure %* |
| --- | --- | --- | --- | --- |
| Binkley 2015^111^ | 55 | NA | 25Gy/1fraction* | 14.3 |
| Filipi 2014^134^ | 67 | NA | 26Gy/1fraction | 18.4 |
| Kalinauskaite 2020^135^ | 25 | NA | 24Gy/1fraction, 25Gy/1fraction* | 17 |
| Siva 2015^129^ | 41 | 59 | 26Gy/1fraction | 10 |

*Most commonly used dose prescription/s in the study; NA: Not provided

eTable 9: **Pulmonary oligometastatic disease from colorectal primary, BED ≥ 100Gy multi fraction SBRT**

| *Study* | *No. of patients* | *Male %* | *Dose/Median BED* | *2-yr Local failure %* |
| --- | --- | --- | --- | --- |
| Baschnagel 2013^109^ | 10 | NA | 60Gy/5fraction* | 20 |
| Binkley 2015^111^ | 10 | NA | 50Gy/4fraction* | 15.7 |
| Franceschini 2017^113^ | 99 | NA | 48Gy/4fraction* | 22 |
| Helou 2017^115^ | 59 | 58 | 48-60Gy/4-5fraction | 23.6 |
| Jingu 2017^116^ | 93 | 65 | Median BED 105.6 | 34.8 |
| Jung 2015^117^ | 50 | NA | Median dose 48Gy/3-4fraction | 20.3 |
| Kim 2009^118^ | 13 | 46 | 39-51Gy/3fraction | 47.3 |
| Kinj 2017^119^ | 53 | 66 | Median BED 180Gy | 21.8 |
| Kobayashi 2020^120^ | 20 | 65 | 54-60Gy/3fraction | 34.2 |
| Li 2019^122^ | 35 | 81 | Median BED 100Gy | 3.7 |
| Nicosia 2020^124^ | 38 | NA | Median BED 105Gy | 20 |
| Oh 2012^126^ | 7 | NA | 60Gy/5fraction* | 20 |
| Takeda 2011^75^ | 15 | 87 | 50Gy/5fraction | 27 |
| Takeda 2016^131^ | 9 | NA | 50-60Gy/5fraction | 4.4 |
| Thibault 2014^132^ | 29 | NA | 48-60Gy/4-5fraction | 24 |
| Wang 2018^84^ | 15 | 67 | 48-60Gy/4-5fraction | 25.4 |
| Yamamoto 2020^133^ | 183 | 61 | Median BED 115.3Gy | 29.7 |

*Most commonly used dose prescription/s in the study; NA: Not provided

eTable 10: **Pulmonary oligometastatic disease from colorectal primary, BED < 100Gy multi fraction SBRT**

| Study | No. of patients | Male % | Dose/Median BED | 2-yr Local failure % |
| --- | --- | --- | --- | --- |
| Li 2019^122^ | 18 | NA | 50Gy/10fraction | 19.4 |
| Okunieff 2006^139^ | 14 | NA | 50-55Gy in 10-11fraction | 17 |
| Qiu 2018^140^ | 65 | 57 | 50Gy/10fraction* | 69.1 |

*Most commonly used dose prescription/s in the study; NA: Not provided

eTable 11: **Pulmonary oligometastatic disease from colorectal primary, BED ≥ 100Gy single fraction SBRT**

| *Study* | *No. of patients* | *Male %* | *Dose/Median BED* | *2-yr Local failure %* |
| --- | --- | --- | --- | --- |
| Osti 2018^136^ | 41 | NA | 120Gy | 25 |

NA: Not provided

eTable 12: **Pulmonary oligometastatic disease from colorectal primary, BED < 100Gy single fraction SBRT**

| *Study* | *No. of patients* | *Male %* | *Dose/Median BED* | *2-yr Local failure %* |
| --- | --- | --- | --- | --- |
| Binkley 2015^111^ | 7 | NA | 24-27Gy/1fraction | 52.6 |

NA: Not provided

**References:**

1.	Abdalmassih M, Bucher O, Rathod S, et al. Clinical Outcomes After Stereotactic Body Radiation Therapy for Early Stage Non-Small Cell Lung Cancer: A Single Institutional Study. *Cureus*. Dec 3 2020;12(12):e11886. doi:10.7759/cureus.11886

2.	Abe T, Ryuno Y, Iino M, et al. A 54 Gy in three fractions of stereotactic body radiotherapy using CyberKnife for T1b-2aN0M0 pathologically confirmed non-small cell lung cancer. *Jpn J Clin Oncol*. Dec 1 2021;51(12):1723-1728. doi:10.1093/jjco/hyab154

3.	Abreu C, Moraes FY, Miranda FA, et al. Stereotactic Body Radiation Therapy for Biopsy-Proven Primary Non-Small-Cell Lung Cancer: Experience of Patients With Inoperable Cancer at a Single Brazilian Institution. *J Glob Oncol*. Jul 2018;4:1-8. doi:10.1200/jgo.18.00020

4.	Alongi F, Nicosia L, Figlia V, et al. A multi-institutional analysis of fractionated versus single-fraction stereotactic body radiotherapy (SBRT) in the treatment of primary lung tumors: a comparison between two antipodal fractionations. *Clinical and Translational Oncology*. 2021;23(10):2133-2140.

5.	Aoki S, Yamashita H, Haga A, et al. Stereotactic body radiotherapy for centrally-located lung tumors with 56 Gy in seven fractions: A retrospective study. *Oncol Lett*. Oct 2018;16(4):4498-4506. doi:10.3892/ol.2018.9188

6.	Arnett ALH, Mou B, Owen D, et al. Long-term Clinical Outcomes and Safety Profile of SBRT for Centrally Located NSCLC. *Adv Radiat Oncol*. Apr-Jun 2019;4(2):422-428. doi:10.1016/j.adro.2019.01.002

7.	Baumann P, Nyman J, Hoyer M, et al. Outcome in a prospective phase II trial of medically inoperable stage I non-small-cell lung cancer patients treated with stereotactic body radiotherapy. *J Clin Oncol*. Jul 10 2009;27(20):3290-6. doi:10.1200/jco.2008.21.5681

8.	Bezjak A, Paulus R, Gaspar LE, et al. Safety and Efficacy of a Five-Fraction Stereotactic Body Radiotherapy Schedule for Centrally Located Non-Small-Cell Lung Cancer: NRG Oncology/RTOG 0813 Trial. *J Clin Oncol*. May 20 2019;37(15):1316-1325. doi:10.1200/jco.18.00622

9.	Bhandari RP, Stanford JD, Packianathan S, et al. Stereotactic Body Radiation Therapy for Stage I Non-Small Cell Lung Cancer: A Retrospective, Single-Center Study of 55 Patients. *Oncology*. 2016;91(4):194-204. doi:10.1159/000447540

10.	Bral S, Gevaert T, Linthout N, et al. Prospective, risk-adapted strategy of stereotactic body radiotherapy for early-stage non-small-cell lung cancer: results of a Phase II trial. *Int J Radiat Oncol Biol Phys*. Aug 1 2011;80(5):1343-9. doi:10.1016/j.ijrobp.2010.04.056

11.	Chang JY, Liu H, Balter P, et al. Clinical outcome and predictors of survival and pneumonitis after stereotactic ablative radiotherapy for stage I non-small cell lung cancer. *Radiat Oncol*. Sep 10 2012;7:152. doi:10.1186/1748-717x-7-152

12.	Chang JY, Mehran RJ, Feng L, et al. Stereotactic ablative radiotherapy for operable stage I non-small-cell lung cancer (revised STARS): long-term results of a single-arm, prospective trial with prespecified comparison to surgery. *Lancet Oncol*. Oct 2021;22(10):1448-1457. doi:10.1016/s1470-2045(21)00401-0

13.	Chaudhuri AA, Tang C, Binkley MS, et al. Stereotactic ablative radiotherapy (SABR) for treatment of central and ultra-central lung tumors. *Lung Cancer*. Jul 2015;89(1):50-6. doi:10.1016/j.lungcan.2015.04.014

14.	Chiang A, Thibault I, Warner A, et al. A comparison between accelerated hypofractionation and stereotactic ablative radiotherapy (SABR) for early-stage non-small cell lung cancer (NSCLC): Results of a propensity score-matched analysis. *Radiother Oncol*. Mar 2016;118(3):478-84. doi:10.1016/j.radonc.2015.12.026

15.	Choi G, Chen G, Mai W, Chen A, Zhu A. Multiple Primary Lung Cancers: Treatment Outcomes After Stereotactic Body Radiotherapy (SBRT). *Cureus*. Jul 2023;15(7):e41319. doi:10.7759/cureus.41319

16.	Cummings MA, Ma SJ, Hermann G, et al. Comparison of Single- and Five-fraction Regimens of Stereotactic Body Radiation Therapy for Peripheral Early-stage Non-small-cell Lung Cancer: A Two-institution Propensity-matched Analysis. *Clin Lung Cancer*. Nov 2018;19(6):511-517. doi:10.1016/j.cllc.2018.07.006

17.	Doupnik NP, Hirmiz K, Hussein AA, Agapito J, Pan M. Early-Stage Non-Small Cell Lung Cancer Stereotactic Body Radiation Therapy Outcomes in a Single Institution. *Cureus*. Feb 2022;14(2):e21878. doi:10.7759/cureus.21878

18.	Dubaere E, Goffaux M, Wanet M, et al. Long term outcome after 48 Gy stereotactic ablative body radiotherapy for peripheral stage I non-small cell lung cancer. *BMC Cancer*. Jun 28 2019;19(1):639. doi:10.1186/s12885-019-5863-2

19.	Factor OB, Vu CC, Schneider JG, et al. Stereotactic body radiation therapy for stage I non-small cell lung cancer: a small academic hospital experience. *Front Oncol*. 2014;4:287. doi:10.3389/fonc.2014.00287

20.	Fakiris AJ, McGarry RC, Yiannoutsos CT, et al. Stereotactic body radiation therapy for early-stage non-small-cell lung carcinoma: four-year results of a prospective phase II study. *Int J Radiat Oncol Biol Phys*. Nov 1 2009;75(3):677-82. doi:10.1016/j.ijrobp.2008.11.042

21.	Fischer-Valuck BW, Boggs H, Katz S, Durci M, Acharya S, Rosen LR. Comparison of stereotactic body radiation therapy for biopsy-proven versus radiographically diagnosed early-stage non-small lung cancer: a single-institution experience. *Tumori*. May-Jun 2015;101(3):287-93. doi:10.5301/tj.5000279

22.	Grills IS, Mangona VS, Welsh R, et al. Outcomes after stereotactic lung radiotherapy or wedge resection for stage I non-small-cell lung cancer. *J Clin Oncol*. Feb 20 2010;28(6):928-35. doi:10.1200/jco.2009.25.0928

23.	Haasbeek CJ, Lagerwaard FJ, Antonisse ME, Slotman BJ, Senan S. Stage I nonsmall cell lung cancer in patients aged > or =75 years: outcomes after stereotactic radiotherapy. *Cancer*. Jan 15 2010;116(2):406-14. doi:10.1002/cncr.24759

24.	Hamamoto Y, Kataoka M, Yamashita M, et al. Local control of metastatic lung tumors treated with SBRT of 48 Gy in four fractions: in comparison with primary lung cancer. *Jpn J Clin Oncol*. Feb 2010;40(2):125-9. doi:10.1093/jjco/hyp129

25.	Hayashi K, Suzuki O, Shiomi H, et al. Stereotactic Ablative Radiotherapy Using CyberKnife for Stage I Non-small-cell Lung Cancer: A Retrospective Analysis. *Anticancer Res*. Jan 2022;42(1):321-327. doi:10.21873/anticanres.15488

26.	Heal C, Ding W, Lamond J, et al. Definitive Treatment of Early-Stage Non-Small Cell Lung Cancer with Stereotactic Ablative Body Radiotherapy in a Community Cancer Center Setting. *Front Oncol*. 2015;5:146. doi:10.3389/fonc.2015.00146

27.	Hobbs CJ, Ko SJ, Paryani NN, et al. Stereotactic Body Radiotherapy for Medically Inoperable Stage I-II Non-Small Cell Lung Cancer: The Mayo Clinic Experience. *Mayo Clin Proc Innov Qual Outcomes*. Mar 2018;2(1):40-48. doi:10.1016/j.mayocpiqo.2017.11.001

28.	Hoyer M RH, Hansen AT, Ohlhuis L, Petersen J, Nellemann H, Berthelsen AK, Grau C, Engelholm SA, Von Der Maase H. Prospective study on stereotactic radiotherapy of limited-stage non–small-cell lung cancer. *International Journal of Radiation Oncology, Biology, Physics*. 2006;63(S396.)

29.	Huang K, Prasad S, Ma SJ, et al. Comparison of Survival Outcomes of Single- and Five-Fraction Schedules of Stereotactic Body Radiation Therapy for Early-Stage Central or Peripheral NSCLC. *Cancers (Basel)*. Mar 8 2023;15(6)doi:10.3390/cancers15061648

30.	Jeppesen SS, Schytte T, Jensen HR, Brink C, Hansen O. Stereotactic body radiation therapy versus conventional radiation therapy in patients with early stage non-small cell lung cancer: an updated retrospective study on local failure and survival rates. *Acta Oncol*. Oct 2013;52(7):1552-8. doi:10.3109/0284186x.2013.813635

31.	Karasawa K, Hayakawa S, Machitori Y, et al. Accelerated Hypofractionated Radiotherapy Versus Stereotactic Body Radiotherapy for the Treatment of Stage I Nonsmall Cell Lung Cancer-A Single Institution Experience With Long-Term Follow-Up. *Technol Cancer Res Treat*. Jan 1 2018;17:1533033818806318. doi:10.1177/1533033818806318

32.	Kim MJ, Yeo SG, Kim ES, Min CK, Se An P. Intensity-modulated stereotactic body radiotherapy for stage I non-small cell lung cancer. *Oncol Lett*. Mar 2013;5(3):840-844. doi:10.3892/ol.2012.1082

33.	Koba A, Hayashi K, Suzuki O, Kawaguchi Y, Ogawa K, Chatani M. Stereotactic body radiotherapy feasibility for patients with peripheral stage I lung cancer and poor pulmonary function. *Oncol Lett*. Mar 2020;19(3):2515-2521. doi:10.3892/ol.2020.11333

34.	Koto M, Takai Y, Ogawa Y, et al. A phase II study on stereotactic body radiotherapy for stage I non-small cell lung cancer. *Radiother Oncol*. Dec 2007;85(3):429-34. doi:10.1016/j.radonc.2007.10.017

35.	Kowalchuk RO, Waters MR, Richardson KM, Spencer KM, Larner JM, Kersh CR. A single institutional experience with central lung stereotactic body radiation therapy demonstrating encouraging results with increased inter-fraction time. *J Thorac Dis*. Feb 2021;13(2):642-652. doi:10.21037/jtd-20-2659

36.	Lagerwaard FJ, Haasbeek CJ, Smit EF, Slotman BJ, Senan S. Outcomes of risk-adapted fractionated stereotactic radiotherapy for stage I non-small-cell lung cancer. *Int J Radiat Oncol Biol Phys*. Mar 1 2008;70(3):685-92. doi:10.1016/j.ijrobp.2007.10.053

37.	Lee S, Song SY, Kim SS, et al. Feasible Optimization of Stereotactic Ablative Radiotherapy Dose by Tumor Size for Stage I Non-small-cell Lung Cancer. *Clin Lung Cancer*. Mar 2018;19(2):e253-e261. doi:10.1016/j.cllc.2017.11.001

38.	Lenglet A, Campeau MP, Mathieu D, et al. Risk-adapted stereotactic ablative radiotherapy for central and ultra-central lung tumours. *Radiother Oncol*. May 2019;134:178-184. doi:10.1016/j.radonc.2019.01.035

39.	Li Q, Swanick CW, Allen PK, et al. Stereotactic ablative radiotherapy (SABR) using 70 Gy in 10 fractions for non-small cell lung cancer: exploration of clinical indications. *Radiother Oncol*. Aug 2014;112(2):256-61. doi:10.1016/j.radonc.2014.07.010

40.	Lischalk JW, Woo SM, Kataria S, et al. Long-term outcomes of stereotactic body radiation therapy (SBRT) with fiducial tracking for inoperable stage I non-small cell lung cancer (NSCLC). *J Radiat Oncol*. 2016;5(4):379-387. doi:10.1007/s13566-016-0273-4

41.	Louie AV, Senan S, Dahele M, Slotman BJ, Verbakel WF. Stereotactic ablative radiation therapy for subcentimeter lung tumors: clinical, dosimetric, and image guidance considerations. *Int J Radiat Oncol Biol Phys*. Nov 15 2014;90(4):843-9. doi:10.1016/j.ijrobp.2014.06.064

42.	Lucas JT, Jr., Kuremsky JG, Soike M, et al. Comparison of accelerated hypofractionation and stereotactic body radiotherapy for Stage 1 and node negative Stage 2 non-small cell lung cancer (NSCLC). *Lung Cancer*. Jul 2014;85(1):59-65. doi:10.1016/j.lungcan.2014.04.003

43.	Ma SJ, Serra LM, Syed YA, Hermann GM, Gomez-Suescun JA, Singh AK. Comparison of Single- and Three-fraction Schedules of Stereotactic Body Radiation Therapy for Peripheral Early-stage Non-Small-cell Lung Cancer. *Clin Lung Cancer*. Mar 2018;19(2):e235-e240. doi:10.1016/j.cllc.2017.10.010

44.	Mancini BR, Park HS, Harder EM, et al. Elderly patients undergoing SBRT for inoperable early-stage NSCLC achieve similar outcomes to younger patients. *Lung Cancer*. Jul 2016;97:22-7. doi:10.1016/j.lungcan.2016.04.011

45.	Matsuo Y, Shibuya K, Nagata Y, et al. Prognostic factors in stereotactic body radiotherapy for non-small-cell lung cancer. *Int J Radiat Oncol Biol Phys*. Mar 15 2011;79(4):1104-11. doi:10.1016/j.ijrobp.2009.12.022

46.	Meng MB, Wang HH, Zaorsky NG, et al. Risk-adapted stereotactic body radiation therapy for central and ultra-central early-stage inoperable non-small cell lung cancer. *Cancer Sci*. Nov 2019;110(11):3553-3564. doi:10.1111/cas.14185

47.	Mihai A, Milano MT, Santos A, et al. Treatment completion, treatment compliance and outcomes of old and very old patients treated by dose adapted stereotactic ablative radiotherapy (SABR) for T1-T3N0M0 non-small cell lung cancer. *J Geriatr Oncol*. May 2019;10(3):442-448. doi:10.1016/j.jgo.2018.10.011

48.	Mix M, Tanny S, Nsouli T, et al. Outcomes Following Stereotactic Body Radiotherapy with Intensity-Modulated Therapy versus Three-Dimensional Conformal Radiotherapy in Early Stage Non-Small Cell Lung Cancer. *Lung Cancer (Auckl)*. 2019;10:151-159. doi:10.2147/lctt.S235713

49.	Miyakawa A, Shibamoto Y, Kosaki K, Hashizume C. Early response and local control of stage I non-small-cell lung cancer after stereotactic radiotherapy: difference by histology. *Cancer Sci*. Jan 2013;104(1):130-4. doi:10.1111/cas.12048

50.	Miyakawa A, Shibamoto Y, Baba F, et al. Stereotactic body radiotherapy for stage I non-small-cell lung cancer using higher doses for larger tumors: results of the second study. *Radiat Oncol*. Sep 11 2017;12(1):152. doi:10.1186/s13014-017-0888-7

51.	Nagata Y, Takayama K, Matsuo Y, et al. Clinical outcomes of a phase I/II study of 48 Gy of stereotactic body radiotherapy in 4 fractions for primary lung cancer using a stereotactic body frame. *Int J Radiat Oncol Biol Phys*. Dec 1 2005;63(5):1427-31. doi:10.1016/j.ijrobp.2005.05.034

52.	Nagata Y, Hiraoka M, Shibata T, et al. Prospective Trial of Stereotactic Body Radiation Therapy for Both Operable and Inoperable T1N0M0 Non-Small Cell Lung Cancer: Japan Clinical Oncology Group Study JCOG0403. *Int J Radiat Oncol Biol Phys*. Dec 1 2015;93(5):989-96. doi:10.1016/j.ijrobp.2015.07.2278

53.	Nath SK, Sandhu AP, Kim D, et al. Locoregional and distant failure following image-guided stereotactic body radiation for early-stage primary lung cancer. *Radiother Oncol*. Apr 2011;99(1):12-7. doi:10.1016/j.radonc.2011.02.006

54.	Ng AW, Tung SY, Wong VY. Hypofractionated stereotactic radiotherapy for medically inoperable stage I non-small cell lung cancer--report on clinical outcome and dose to critical organs. *Radiother Oncol*. Apr 2008;87(1):24-8. doi:10.1016/j.radonc.2008.02.015

55.	Nittala MR, Duggar WN, Mundra E, et al. Single Institution Experience of Stereotactic Body Radiation Therapy in Non-small Cell Lung Cancer: Comparison of Two Dose Regimes and a Perspective on Ideal Dose Regimens. *Cureus*. Oct 2021;13(10):e18862. doi:10.7759/cureus.18862

56.	Nyman J, Hallqvist A, Lund J, et al. SPACE - A randomized study of SBRT vs conventional fractionated radiotherapy in medically inoperable stage I NSCLC. *Radiother Oncol*. Oct 2016;121(1):1-8. doi:10.1016/j.radonc.2016.08.015

57.	Olsen JR RC, El Naqa I, Creach KM, Drzymala RE, Bloch C, Parikh PJ, Bradley JD. Dose–Response For Stereotactic Body Radiotherapy In Early-Stage Non–Small-Cell Lung Cancer. *International Journal of Radiation Oncology Biology Physics*. 2011;81(4):299-303.

58.	Onimaru R, Fujino M, Yamazaki K, et al. Steep dose-response relationship for stage I non-small-cell lung cancer using hypofractionated high-dose irradiation by real-time tumor-tracking radiotherapy. *Int J Radiat Oncol Biol Phys*. Feb 1 2008;70(2):374-81. doi:10.1016/j.ijrobp.2007.06.043

59.	Onishi H, Shirato H, Nagata Y, et al. Stereotactic body radiotherapy (SBRT) for operable stage I non-small-cell lung cancer: can SBRT be comparable to surgery? *Int J Radiat Oncol Biol Phys*. Dec 1 2011;81(5):1352-8. doi:10.1016/j.ijrobp.2009.07.1751

60.	Patel M, Colvin T, Kirkland RS, et al. Reduced Margin Stereotactic Body Radiation Therapy for Early Stage Non-Small Cell Lung Cancers. *Cureus*. Jun 14 2020;12(6):e8618. doi:10.7759/cureus.8618

61.	Raman S, Yau V, Pineda S, et al. Ultracentral Tumors Treated With Stereotactic Body Radiotherapy: Single-Institution Experience. *Clin Lung Cancer*. Sep 2018;19(5):e803-e810. doi:10.1016/j.cllc.2018.06.001

62.	Ricardi U, Filippi AR, Guarneri A, et al. Stereotactic body radiation therapy for early stage non-small cell lung cancer: results of a prospective trial. *Lung Cancer*. Apr 2010;68(1):72-7. doi:10.1016/j.lungcan.2009.05.007

63.	Ricardi U, Frezza G, Filippi AR, et al. Stereotactic Ablative Radiotherapy for stage I histologically proven non-small cell lung cancer: an Italian multicenter observational study. *Lung Cancer*. Jun 2014;84(3):248-53. doi:10.1016/j.lungcan.2014.02.015

64.	Román A, Perez-Rozos A, Otero A, et al. Efficacy and safety of a simplified SBRT regimen for central and peripheral lung tumours. *Clin Transl Oncol*. Jan 2020;22(1):144-150. doi:10.1007/s12094-019-02119-6

65.	Ryuno Y, Abe T, Iino M, et al. High-dose stereotactic body radiotherapy using CyberKnife® for stage I peripheral lung cancer: a single-center retrospective study. *Radiat Oncol*. Jul 19 2022;17(1):128. doi:10.1186/s13014-022-02094-3

66.	Scotti V, Bruni A, Francolini G, et al. Stereotactic Ablative Radiotherapy as an Alternative to Lobectomy in Patients With Medically Operable Stage I NSCLC: A Retrospective, Multicenter Analysis. *Clin Lung Cancer*. Jan 2019;20(1):e53-e61. doi:10.1016/j.cllc.2018.09.003

67.	Shintani T, Matsuo Y, Iizuka Y, Mitsuyoshi T, Mizowaki T. A Retrospective Long-term Follow-up Study of Stereotactic Body Radiation Therapy for Non-Small Cell Lung Cancer From a Single Institution: Incidence of Late Local Recurrence. *Int J Radiat Oncol Biol Phys*. Apr 1 2018;100(5):1228-1236. doi:10.1016/j.ijrobp.2018.01.050

68.	Shirata Y, Jingu K, Koto M, et al. Prognostic factors for local control of stage I non-small cell lung cancer in stereotactic radiotherapy: a retrospective analysis. *Radiat Oncol*. Oct 31 2012;7:182. doi:10.1186/1748-717x-7-182

69.	Singh AK G-SJ, Stephans KL, Bogart JA, Hermann GM, Tian L, Groman A, Videtic GM. One versus three fractions of stereotactic body radiation therapy for peripheral stage I to II non-small cell lung cancer: a randomized, multi-institution, phase 2 trial. *International Journal of Radiation Oncology* Biology* Physics*. 2019;105(4):752-9.

70.	Song SY, Choi W, Shin SS, et al. Fractionated stereotactic body radiation therapy for medically inoperable stage I lung cancer adjacent to central large bronchus. *Lung Cancer*. Oct 2009;66(1):89-93. doi:10.1016/j.lungcan.2008.12.016

71.	Song X, Zhao L, Jiang N, et al. Long-term outcomes in patients with central and ultracentral non-small cell lung cancer treated with stereotactic body radiotherapy: single-institution experience. *Curr Probl Cancer*. Jun 2023;47(3):100956. doi:10.1016/j.currproblcancer.2023.100956

72.	Stephans KL, Woody NM, Reddy CA, et al. Tumor Control and Toxicity for Common Stereotactic Body Radiation Therapy Dose-Fractionation Regimens in Stage I Non-Small Cell Lung Cancer. *Int J Radiat Oncol Biol Phys*. Feb 1 2018;100(2):462-469. doi:10.1016/j.ijrobp.2017.10.037

73.	Sun B, Brooks ED, Komaki RU, et al. 7-year follow-up after stereotactic ablative radiotherapy for patients with stage I non-small cell lung cancer: Results of a phase 2 clinical trial. *Cancer*. Aug 15 2017;123(16):3031-3039. doi:10.1002/cncr.30693

74.	Takeda A, Sanuki N, Kunieda E, et al. Stereotactic body radiotherapy for primary lung cancer at a dose of 50 Gy total in five fractions to the periphery of the planning target volume calculated using a superposition algorithm. *Int J Radiat Oncol Biol Phys*. Feb 1 2009;73(2):442-8. doi:10.1016/j.ijrobp.2008.04.043

75.	Takeda A, Kunieda E, Ohashi T, Aoki Y, Koike N, Takeda T. Stereotactic body radiotherapy (SBRT) for oligometastatic lung tumors from colorectal cancer and other primary cancers in comparison with primary lung cancer. *Radiother Oncol*. Nov 2011;101(2):255-9. doi:10.1016/j.radonc.2011.05.033

76.	Taremi M, Hope A, Dahele M, et al. Stereotactic body radiotherapy for medically inoperable lung cancer: prospective, single-center study of 108 consecutive patients. *Int J Radiat Oncol Biol Phys*. Feb 1 2012;82(2):967-73. doi:10.1016/j.ijrobp.2010.12.039

77.	Timmerman R, Paulus R, Galvin J, et al. Stereotactic body radiation therapy for inoperable early stage lung cancer. *Jama*. Mar 17 2010;303(11):1070-6. doi:10.1001/jama.2010.261

78.	Timmerman RD, Paulus R, Pass HI, et al. Stereotactic Body Radiation Therapy for Operable Early-Stage Lung Cancer: Findings From the NRG Oncology RTOG 0618 Trial. *JAMA Oncol*. Sep 1 2018;4(9):1263-1266. doi:10.1001/jamaoncol.2018.1251

79.	Trakul N, Chang CN, Harris J, et al. Tumor volume-adapted dosing in stereotactic ablative radiotherapy of lung tumors. *Int J Radiat Oncol Biol Phys*. Sep 1 2012;84(1):231-7. doi:10.1016/j.ijrobp.2011.10.071

80.	Turzer M, Brustugun OT, Waldeland E, Helland A. Stereotactic body radiation therapy is effective and safe in patients with early-stage non-small cell lung cancer with low performance status and severe comorbidity. *Case Rep Oncol*. Jan 21 2011;4(1):25-34. doi:10.1159/000324113

81.	van der Voort van Zyp NC, Prévost JB, Hoogeman MS, et al. Stereotactic radiotherapy with real-time tumor tracking for non-small cell lung cancer: clinical outcome. *Radiother Oncol*. Jun 2009;91(3):296-300. doi:10.1016/j.radonc.2009.02.011

82.	Verstegen NE, Lagerwaard FJ, Haasbeek CJ, Slotman BJ, Senan S. Outcomes of stereotactic ablative radiotherapy following a clinical diagnosis of stage I NSCLC: comparison with a contemporaneous cohort with pathologically proven disease. *Radiother Oncol*. Nov 2011;101(2):250-4. doi:10.1016/j.radonc.2011.09.017

83.	Videtic G, Paulus R, Singh A, et al. Long-term Follow-up on NRG Oncology RTOG 0915 (NCCTG N0927): A Randomized Phase 2 Study Comparing 2 Stereotactic Body Radiation Therapy Schedules for Medically Inoperable Patients With Stage I Peripheral Non-Small Cell Lung Cancer. *International Journal of Radiation Oncology Biology Physics*. 2019;103(5):1077-1084.

84.	Wang X, Zamdborg L, Ye H, Grills IS, Yan D. A matched-pair analysis of stereotactic body radiotherapy (SBRT) for oligometastatic lung tumors from colorectal cancer versus early stage non-small cell lung cancer. *BMC Cancer*. Oct 10 2018;18(1):962. doi:10.1186/s12885-018-4865-9

85.	Watanabe K, Katsui K, Sugiyama S, et al. Lung stereotactic body radiation therapy for elderly patients aged ≥ 80 years with pathologically proven early-stage non-small cell lung cancer: a retrospective cohort study. *Radiat Oncol*. Feb 23 2021;16(1):39. doi:10.1186/s13014-021-01769-7

86.	Wegner RE, Ahmed N, Hasan S, Schumacher LY, Van Deusen M, Colonias A. SBRT for early stage lung cancer: outcomes from biopsy-proven and empirically treated lesions. *Lung Cancer Manag*. Mar 2018;7(1):Lmt01. doi:10.2217/lmt-2018-0006

87.	Xia T, Li H, Sun Q, et al. Promising clinical outcome of stereotactic body radiation therapy for patients with inoperable Stage I/II non-small-cell lung cancer. *Int J Radiat Oncol Biol Phys*. Sep 1 2006;66(1):117-25. doi:10.1016/j.ijrobp.2006.04.013

88.	Yamashita H, Haga A, Takahashi W, et al. Volumetric modulated arc therapy for lung stereotactic radiation therapy can achieve high local control rates. *Radiat Oncol*. Nov 11 2014;9:243. doi:10.1186/s13014-014-0243-1

89.	Yang D, Cui J, Zhao J, et al. Stereotactic ablative radiotherapy of 60 Gy in eight fractions is safe for ultracentral non-small cell lung cancer. *Thorac Cancer*. Mar 2020;11(3):754-761. doi:10.1111/1759-7714.13335

90.	Yaprak G, Ozan Seseogullari O, Dogan Akaslan B, Isik N. Is stereotactic body radiotherapy an alternative to surgery in early stage non small cell lung cancer? *J buon*. Jul-Aug 2019;24(4):1619-1625.

91.	Yoshitake T, Nakamura K, Shioyama Y, et al. Stereotactic body radiation therapy for primary lung cancers clinically diagnosed without pathological confirmation: a single-institution experience. *Int J Clin Oncol*. Feb 2015;20(1):53-8. doi:10.1007/s10147-014-0698-y

92.	Ahmed N, Hasan S, Schumacher L, Colonias A, Wegner RE. Stereotactic body radiotherapy for central lung tumors: Finding the balance between safety and efficacy in the "no fly" zone. *Thorac Cancer*. Oct 2018;9(10):1211-1214. doi:10.1111/1759-7714.12764

93.	Kundu S, Mathew A, Munshi A, Prabhash K, Pramesh CS, Agarwal JP. Stereotactic body radiotherapy in early stage non-small cell lung cancer: first experience from an Indian Centre. *Indian J Cancer*. Jul-Sep 2013;50(3):227-32. doi:10.4103/0019-509x.118739

94.	Lodeweges JE, van Rossum PSN, Bartels M, et al. Ultra-central lung tumors: safety and efficacy of protracted stereotactic body radiotherapy. *Acta Oncol*. Aug 2021;60(8):1061-1068. doi:10.1080/0284186x.2021.1942545

95.	Nestle U, Adebahr S, Kaier K, et al. Quality of life after pulmonary stereotactic fractionated radiotherapy (SBRT): Results of the phase II STRIPE trial. *Radiother Oncol*. Jul 2020;148:82-88. doi:10.1016/j.radonc.2020.03.018

96.	Schanne DH, Nestle U, Allgäuer M, et al. Stereotactic body radiotherapy for centrally located stage I NSCLC: a multicenter analysis. *Strahlenther Onkol*. Feb 2015;191(2):125-32. doi:10.1007/s00066-014-0739-5

97.	Testolin A, Favretto MS, Cora S, Cavedon C. Stereotactic body radiation therapy for a new lung cancer arising after pneumonectomy: dosimetric evaluation and pulmonary toxicity. *Br J Radiol*. 2015;88(1055):20150228. doi:10.1259/bjr.20150228

98.	Wulf J HU, Oppitz U, Thiele W, Mueller G, Flentje M. Stereotactic radiotherapy for primary lung cancer and pulmonary metastases: a noninvasive treatment approach in medically inoperable patients. *International Journal of Radiation Oncology* Biology* Physics*. 2004;60(1):186-96.

99.	Zimmermann FB, Geinitz H, Schill S, et al. Stereotactic hypofractionated radiation therapy for stage I non-small cell lung cancer. *Lung Cancer*. Apr 2005;48(1):107-14. doi:10.1016/j.lungcan.2004.10.015

100.	Fritz P, Kraus HJ, Mühlnickel W, et al. Stereotactic, single-dose irradiation of stage I non-small cell lung cancer and lung metastases. *Radiat Oncol*. Aug 20 2006;1:30. doi:10.1186/1748-717x-1-30

101.	Fritz P, Kraus HJ, Blaschke T, et al. Stereotactic, high single-dose irradiation of stage I non-small cell lung cancer (NSCLC) using four-dimensional CT scans for treatment planning. *Lung Cancer*. May 2008;60(2):193-9. doi:10.1016/j.lungcan.2007.10.005

102.	Iovoli AJ, Prasad S, Ma SJ, et al. Long-Term Survival and Failure Outcomes of Single-Fraction Stereotactic Body Radiation Therapy in Early Stage NSCLC. *JTO Clin Res Rep*. Dec 2023;4(12):100598. doi:10.1016/j.jtocrr.2023.100598

103.	Manyam BV, Videtic GMM, Verdecchia K, Reddy CA, Woody NM, Stephans KL. Effect of Tumor Location and Dosimetric Predictors for Chest Wall Toxicity in Single-Fraction Stereotactic Body Radiation Therapy for Stage I Non-Small Cell Lung Cancer. *Pract Radiat Oncol*. Mar 2019;9(2):e187-e195. doi:10.1016/j.prro.2018.11.011

104.	Nicosia L, Reverberi C, Agolli L, et al. Long term results of single high dose Stereotactic Body Radiotherapy in the treatment of primary lung tumors. *Sci Rep*. Oct 29 2019;9(1):15498. doi:10.1038/s41598-019-51900-8

105.	Videtic GMM, Reddy CA, Woody NM, Stephans KL. Ten-Year Experience in Implementing Single-Fraction Lung SBRT for Medically Inoperable Early-Stage Lung Cancer. *Int J Radiat Oncol Biol Phys*. Oct 1 2021;111(2):436-442. doi:10.1016/j.ijrobp.2021.05.116

106.	Hof H, Herfarth KK, Münter M, et al. Stereotactic single-dose radiotherapy of stage I non-small-cell lung cancer (NSCLC). *Int J Radiat Oncol Biol Phys*. Jun 1 2003;56(2):335-41. doi:10.1016/s0360-3016(02)04504-2

107.	Hof H, Muenter M, Oetzel D, Hoess A, Debus J, Herfarth K. Stereotactic single-dose radiotherapy (radiosurgery) of early stage nonsmall-cell lung cancer (NSCLC). *Cancer*. Jul 1 2007;110(1):148-55. doi:10.1002/cncr.22763

108.	Le QT, Loo BW, Ho A, et al. Results of a phase I dose-escalation study using single-fraction stereotactic radiotherapy for lung tumors. *J Thorac Oncol*. Oct 2006;1(8):802-9.

109.	Baschnagel AM, Mangona VS, Robertson JM, Welsh RJ, Kestin LL, Grills IS. Lung metastases treated with image-guided stereotactic body radiation therapy. *Clin Oncol (R Coll Radiol)*. Apr 2013;25(4):236-41. doi:10.1016/j.clon.2012.12.005

110.	Berkovic P, Gulyban A, Defraene G, et al. Stereotactic robotic body radiotherapy for patients with oligorecurrent pulmonary metastases. *BMC Cancer*. May 8 2020;20(1):402. doi:10.1186/s12885-020-06906-1

111.	Binkley MS, Trakul N, Jacobs LR, et al. Colorectal Histology Is Associated With an Increased Risk of Local Failure in Lung Metastases Treated With Stereotactic Ablative Radiation Therapy. *Int J Radiat Oncol Biol Phys*. Aug 1 2015;92(5):1044-1052. doi:10.1016/j.ijrobp.2015.04.004

112.	Dohopolski MJ, Horne Z, Clump D, Burton SA, Heron DE. Stereotactic Body Radiation Therapy for Pulmonary Oligometastases Arising from Non-lung Primaries in Patients Without Extrapulmonary Disease. *Cureus*. Feb 7 2018;10(2):e2167. doi:10.7759/cureus.2167

113.	Franceschini D, Cozzi L, De Rose F, et al. Role of stereotactic body radiation therapy for lung metastases from radio-resistant primary tumours. *J Cancer Res Clin Oncol*. Jul 2017;143(7):1293-1299. doi:10.1007/s00432-017-2373-y

114.	García-Cabezas S, Bueno C, Rivin E, Roldán JM, Palacios-Eito A. Lung metastases in oligometastatic patients: outcome with stereotactic body radiation therapy (SBRT). *Clin Transl Oncol*. Aug 2015;17(8):668-72. doi:10.1007/s12094-015-1291-1

115.	Helou J, Thibault I, Poon I, et al. Stereotactic Ablative Radiation Therapy for Pulmonary Metastases: Histology, Dose, and Indication Matter. *Int J Radiat Oncol Biol Phys*. Jun 1 2017;98(2):419-427. doi:10.1016/j.ijrobp.2017.02.093

116.	Jingu K, Matsuo Y, Onishi H, et al. Dose Escalation Improves Outcome in Stereotactic Body Radiotherapy for Pulmonary Oligometastases from Colorectal Cancer. *Anticancer Res*. May 2017;37(5):2709-2713. doi:10.21873/anticanres.11621

117.	Jung J, Song SY, Kim JH, et al. Clinical efficacy of stereotactic ablative radiotherapy for lung metastases arising from colorectal cancer. *Radiat Oncol*. Nov 21 2015;10:238. doi:10.1186/s13014-015-0546-x

118.	Kim MS, Yoo SY, Cho CK, et al. Stereotactic body radiation therapy using three fractions for isolated lung recurrence from colorectal cancer. *Oncology*. 2009;76(3):212-9. doi:10.1159/000201932

119.	Kinj R, Bondiau PY, François E, et al. Radiosensitivity of Colon and Rectal Lung Oligometastasis Treated With Stereotactic Ablative Radiotherapy. *Clin Colorectal Cancer*. Sep 2017;16(3):e211-e220. doi:10.1016/j.clcc.2016.08.003

120.	Kobayashi N, Abe T, Noda SE, et al. Stereotactic Body Radiotherapy for Pulmonary Oligometastasis from Colorectal Cancer. *In Vivo*. Sep-Oct 2020;34(5):2991-2996. doi:10.21873/invivo.12130

121.	Lee YH, Kang KM, Choi HS, et al. Comparison of stereotactic body radiotherapy versus metastasectomy outcomes in patients with pulmonary metastases. *Thorac Cancer*. Dec 2018;9(12):1671-1679. doi:10.1111/1759-7714.12880

122.	Li S, Dong D, Geng J, et al. Prognostic Factors and Optimal Response Interval for Stereotactic Body Radiotherapy in Patients With Lung Oligometastases or Oligoprogression From Colorectal Cancer. *Front Oncol*. 2019;9:1080. doi:10.3389/fonc.2019.01080

123.	Navarria P, Ascolese AM, Tomatis S, et al. Stereotactic body radiotherapy (sbrt) in lung oligometastatic patients: role of local treatments. *Radiat Oncol*. Apr 2 2014;9(1):91. doi:10.1186/1748-717x-9-91

124.	Nicosia L, Cuccia F, Mazzola R, et al. Disease course of lung oligometastatic colorectal cancer treated with stereotactic body radiotherapy. *Strahlenther Onkol*. Sep 2020;196(9):813-820. doi:10.1007/s00066-020-01627-7

125.	Norihisa Y, Nagata Y, Takayama K, et al. Stereotactic body radiotherapy for oligometastatic lung tumors. *Int J Radiat Oncol Biol Phys*. Oct 1 2008;72(2):398-403. doi:10.1016/j.ijrobp.2008.01.002

126.	Oh D, Ahn YC, Seo JM, et al. Potentially curative stereotactic body radiation therapy (SBRT) for single or oligometastasis to the lung. *Acta Oncol*. May 2012;51(5):596-602. doi:10.3109/0284186x.2012.681698

127.	Ricco A, Davis J, Rate W, et al. Lung metastases treated with stereotactic body radiotherapy: the RSSearch® patient Registry's experience. *Radiat Oncol*. Feb 1 2017;12(1):35. doi:10.1186/s13014-017-0773-4

128.	Rusthoven KE, Kavanagh BD, Burri SH, et al. Multi-institutional phase I/II trial of stereotactic body radiation therapy for lung metastases. *J Clin Oncol*. Apr 1 2009;27(10):1579-84. doi:10.1200/jco.2008.19.6386

129.	Siva S, Kirby K, Caine H, et al. Comparison of Single-fraction and Multi-fraction Stereotactic Radiotherapy for Patients with 18F-fluorodeoxyglucose Positron Emission Tomography-staged Pulmonary Oligometastases. *Clin Oncol (R Coll Radiol)*. Jun 2015;27(6):353-61. doi:10.1016/j.clon.2015.01.004

130.	Siva S, Bressel M, Mai T, et al. Single-Fraction vs Multifraction Stereotactic Ablative Body Radiotherapy for Pulmonary Oligometastases (SAFRON II): The Trans Tasman Radiation Oncology Group 13.01 Phase 2 Randomized Clinical Trial. *JAMA Oncol*. Oct 1 2021;7(10):1476-1485. doi:10.1001/jamaoncol.2021.2939

131.	Takeda A, Sanuki N, Tsurugai Y, Oku Y, Aoki Y. Stereotactic body radiotherapy for patients with oligometastases from colorectal cancer: risk-adapted dose prescription with a maximum dose of 83-100 Gy in five fractions. *J Radiat Res*. Jul 2016;57(4):400-5. doi:10.1093/jrr/rrw029

132.	Thibault I, Poon I, Yeung L, et al. Predictive factors for local control in primary and metastatic lung tumours after four to five fraction stereotactic ablative body radiotherapy: a single institution's comprehensive experience. *Clin Oncol (R Coll Radiol)*. Nov 2014;26(11):713-9. doi:10.1016/j.clon.2014.06.018

133.	Yamamoto T, Niibe Y, Matsumoto Y, et al. Analyses of local control and survival after stereotactic body radiotherapy for pulmonary oligometastases from colorectal adenocarcinoma. *J Radiat Res*. Nov 16 2020;61(6):935-944. doi:10.1093/jrr/rraa071

134.	Filippi AR, Badellino S, Guarneri A, et al. Outcomes of single fraction stereotactic ablative radiotherapy for lung metastases. *Technol Cancer Res Treat*. Feb 2014;13(1):37-45. doi:10.7785/tcrt.2012.500355

135.	Kalinauskaite GG, Tinhofer, II, Kufeld MM, et al. Radiosurgery and fractionated stereotactic body radiotherapy for patients with lung oligometastases. *BMC Cancer*. May 11 2020;20(1):404. doi:10.1186/s12885-020-06892-4

136.	Osti MF, Agolli L, Valeriani M, et al. 30 Gy single dose stereotactic body radiation therapy (SBRT): Report on outcome in a large series of patients with lung oligometastatic disease. *Lung Cancer*. Aug 2018;122:165-170. doi:10.1016/j.lungcan.2018.06.018

137.	Chai G, Yin Y, Zhou X, et al. Pulmonary oligometastases treated by stereotactic body radiation therapy (SBRT): a single institution's experience. *Transl Lung Cancer Res*. Aug 2020;9(4):1496-1506. doi:10.21037/tlcr-20-867

138.	Lischalk JW, Malik RM, Collins SP, Collins BT, Matus IA, Anderson ED. Stereotactic body radiotherapy (SBRT) for high-risk central pulmonary metastases. *Radiat Oncol*. Feb 27 2016;11:28. doi:10.1186/s13014-016-0608-8

139.	Okunieff P, Petersen AL, Philip A, et al. Stereotactic Body Radiation Therapy (SBRT) for lung metastases. *Acta Oncol*. 2006;45(7):808-17. doi:10.1080/02841860600908954

140.	Qiu H, Katz AW, Chowdhry AK, et al. Stereotactic Body Radiotherapy for Lung Metastases from Colorectal Cancer: Prognostic Factors for Disease Control and Survival. *Am J Clin Oncol*. Jan 2018;41(1):53-58. doi:10.1097/coc.0000000000000220

**eFig 1(a)	Primary Lung Cancer BED ≥ 100Gy Multi Fraction**

| **N** | **2yr LR** | **LCI** | **UCI** |
| --- | --- | --- | --- |
| **158** | **13.00** | **8.59** | **19.20** |
| **26** | **1.54** | **0.07** | **26.20** |
| **54** | **10.90** | **4.94** | **22.35** |
| **29** | **3.80** | **0.59** | **20.95** |
| **35** | **4.00** | **0.76** | **18.43** |
| **103** | **5.00** | **2.12** | **11.32** |
| **57** | **5.40** | **1.78** | **15.26** |
| **71** | **11.30** | **5.76** | **20.99** |
| **55** | **9.00** | **3.78** | **19.94** |
| **40** | **16.00** | **7.56** | **30.73** |
| **80** | **3.68** | **1.18** | **10.90** |
| **130** | **1.50** | **0.37** | **5.89** |
| **68** | **10.00** | **4.79** | **19.70** |
| **57** | **4.40** | **1.28** | **14.03** |
| **38** | **6.70** | **1.97** | **20.39** |
| **98** | **18.00** | **11.59** | **26.87** |
| **44** | **6.00** | **1.81** | **18.13** |
| **189** | **15.00** | **10.58** | **20.83** |
| **74** | **13.00** | **7.05** | **22.73** |
| **70** | **8.10** | **3.60** | **17.22** |
| **88** | **7.00** | **3.21** | **14.58** |
| **58** | **4.00** | **1.11** | **13.42** |
| **193** | **5.20** | **2.82** | **9.38** |
| **52** | **12.00** | **5.58** | **23.94** |
| **74** | **7.16** | **3.09** | **15.73** |
| **153** | **8.10** | **4.70** | **13.61** |
| **100** | **6.00** | **2.72** | **12.72** |
| **282** | **5.00** | **2.99** | **8.25** |
| **40** | **15.00** | **6.90** | **29.59** |
| **100** | **19.10** | **12.54** | **27.99** |
| **56** | **16.60** | **8.96** | **28.69** |
| **16** | **9.00** | **1.75** | **35.40** |
| **95** | **22.00** | **14.79** | **31.43** |
| **31** | **28.20** | **15.23** | **46.20** |
| **98** | **23.10** | **15.81** | **32.46** |
| **206** | **7.00** | **4.22** | **11.39** |
| **137** | **18.60** | **12.94** | **26.00** |
| **169** | **11.70** | **7.65** | **17.48** |
| **82** | **3.78** | **1.25** | **10.89** |
| **61** | **1.90** | **0.31** | **10.85** |
| **9** | **4.44** | **0.19** | **52.55** |
| **81** | **7.10** | **3.17** | **15.14** |
| **94** | **13.80** | **8.18** | **22.34** |
| **251** | **12.79** | **9.19** | **17.52** |
| **101** | **8.31** | **4.28** | **15.52** |
| **80** | **4.00** | **1.34** | **11.31** |
| **200** | **11.33** | **7.62** | **16.51** |
| **297** | **5.70** | **3.57** | **8.99** |
| **71** | **7.50** | **3.24** | **16.39** |
| **91** | **11.44** | **6.34** | **19.76** |
| **164** | **10.94** | **7.00** | **16.71** |
| **45** | **3.60** | **0.77** | **15.20** |
| **48** | **5.00** | **1.42** | **16.16** |
| **20** | **5.30** | **0.79** | **28.36** |
| **114** | **16.74** | **10.95** | **24.74** |
| **49** | **9.25** | **3.73** | **21.13** |
| **122** | **8.20** | **4.47** | **14.57** |
| **28** | **10.10** | **3.18** | **27.75** |
| **87** | **4.71** | **1.80** | **11.76** |
| **152** | **5.00** | **2.47** | **9.84** |
| **152** | **2.87** | **1.13** | **7.11** |
| **196** | **9.10** | **5.80** | **14.01** |
| **62** | **8.31** | **3.55** | **18.25** |
| **60** | **9.74** | **4.39** | **20.21** |
| **96** | **3.60** | **1.26** | **9.85** |
| **93** | **9.68** | **5.11** | **17.57** |
| **216** | **13.20** | **9.30** | **18.40** |
| **74** | **1.60** | **0.26** | **9.09** |
| **49** | **2.90** | **0.56** | **13.68** |
| **40** | **7.94** | **2.67** | **21.34** |
| **32** | **16.10** | **6.96** | **33.00** |
| **519** | **12.63** | **10.04** | **15.78** |
| **65** | **2.99** | **0.65** | **12.70** |
| **183** | **7.00** | **1.64** | **25.35** |
| **63** | **5.81** | **2.68** | **12.14** |
| **108** | **8.80** | **4.72** | **15.81** |
| **26** | **4.00** | **0.58** | **22.86** |
| **55** | **1.61** | **0.20** | **11.79** |
| **55** | **7.90** | **3.12** | **18.60** |
| **36** | **1.11** | **0.05** | **20.23** |
| **70** | **6.83** | **2.81** | **15.65** |
| **591** | **5.50** | **3.93** | **7.65** |
| **45** | **2.20** | **0.31** | **14.15** |
| **46** | **2.80** | **0.50** | **14.24** |
| **65** | **5.50** | **1.96** | **14.46** |
| **196** | **6.00** | **3.42** | **10.32** |
| **43** | **3.36** | **0.66** | **15.44** |
| **51** | **8.00** | **3.06** | **19.30** |
| **21** | **7.10** | **1.42** | **28.78** |
| **52** | **8.00** | **3.09** | **19.15** |
| **88** | **4.60** | **1.75** | **11.56** |
| **9119** | **9.96** | **9.32** | **10.65** |
| **9119** | **8.56** | **7.58** | **9.65** |

**eFig 1(b)	Primary Lung Cancer BED ≥ 100Gy Single Fraction**

| **N** | **2y LR** | **LCI** | **UCI** |
| --- | --- | --- | --- |
| **44** | **12.20** | **5.33** | **25.53** |
| **65** | **8.00** | **3.43** | **17.56** |
| **40** | **4.90** | **1.21** | **17.80** |
| **33** | **1.21** | **0.05** | **21.71** |
| **191** | **6.40** | **3.69** | **10.88** |
| **263** | **8.00** | **5.28** | **11.95** |
| **139** | **7.30** | **3.99** | **12.98** |
| **65** | **12.20** | **6.20** | **22.60** |
| **44** | **9.21** | **3.53** | **21.99** |
| **49** | **12.82** | **5.98** | **25.36** |
| **84** | **13.10** | **7.41** | **22.13** |
| **39** | **2.60** | **0.37** | **16.10** |
| **229** | **7.30** | **4.57** | **11.47** |
| **1285** | **8.068** | **6.622** | **9.797** |
| **11285** | **8.068** | **6.622** | **9.797** |

**eFig 1(c)	Primary Lung Cancer BED < 100Gy Multiple Fraction**

|  | **N** | **2yr LR** | **LCI** | **UCI** |
| --- | --- | --- | --- | --- |
| **18** | | **13.00** | **3.65** | **37.12** |
| **8** | | **5.00** | **0.22** | **55.85** |
| **72** | | **15.00** | **8.46** | **25.21** |
| **56** | | **7.30** | **2.80** | **17.73** |
| **8** | | **50.00** | **20.01** | **79.99** |
| **13** | | **56.80** | **30.50** | **79.76** |
| **90** | | **36.00** | **26.78** | **46.38** |
| **7** | | **42.90** | **14.40** | **77.05** |
| **5** | | **20.00** | **2.72** | **69.11** |
| **20** | | **5.00** | **0.70** | **28.22** |
| **68** | | **12.00** | **6.16** | **22.08** |
| **365** | | **23.85** | **19.27** | **29.14** |
| **365** | | **21.01** | **12.38** | **33.35** |

| **N** | **2yr LR** | **LCI** | **UCI** |
| --- | --- | --- | --- |
| **10** | **28.90** | **9.38** | **61.47** |
| **10** | **50.00** | **22.45** | **77.55** |
| **32** | **31.00** | **14.83** | **53.68** |
| **5** | **40.00** | **10.02** | **79.96** |
| **36** | **17.80** | **8.44** | **33.72** |
| **81** | **28.84** | **19.71** | **40.08** |
| **81** | **29.26** | **19.48** | **41.43** |

**eFig 1(d)	Primary Lung Cancer BED < 100Gy Single Fraction**

**eFig 2(a)	Metastatic Lung Cancer BED ≥ 100Gy Multi Fraction (any primary histology)**

| **N** | **2yr LR** | **LCI** | **UCI** |
| --- | --- | --- | --- |
| **32** | **8.00** | **2.37** | **23.77** |
| **104** | **20.00** | **13.39** | **28.79** |
| **23** | **7.96** | **1.87** | **28.13** |
| **105** | **6.00** | **2.77** | **12.50** |
| **200** | **15.10** | **10.78** | **20.76** |
| **44** | **13.30** | **6.04** | **26.80** |
| **10** | **75.00** | **41.76** | **92.62** |
| **120** | **15.20** | **9.82** | **22.78** |
| **22** | **4.90** | **0.74** | **26.31** |
| **50** | **27.10** | **16.61** | **40.95** |
| **13** | **47.30** | **23.20** | **72.72** |
| **53** | **21.80** | **12.68** | **34.86** |
| **20** | **34.20** | **17.10** | **56.70** |
| **21** | **24.80** | **10.91** | **47.03** |
| **35** | **3.70** | **0.66** | **18.18** |
| **11** | **3.64** | **0.16** | **47.00** |
| **76** | **11.00** | **5.68** | **20.23** |
| **38** | **20.00** | **10.15** | **35.63** |
| **34** | **10.00** | **3.50** | **25.41** |
| **57** | **2.50** | **0.48** | **11.91** |
| **178** | **26.30** | **20.36** | **33.25** |
| **31** | **13.58** | **5.32** | **30.51** |
| **38** | **4.00** | **0.82** | **17.43** |
| **24** | **8.00** | **1.95** | **27.54** |
| **45** | **20.00** | **10.75** | **34.17** |
| **34** | **18.00** | **8.38** | **34.49** |
| **9** | **4.44** | **0.19** | **52.55** |
| **53** | **15.90** | **8.30** | **28.30** |
| **4** | **8.00** | **0.23** | **76.31** |
| **15** | **28.00** | **11.19** | **54.55** |
| **183** | **29.65** | **23.48** | **36.66** |
| **16** | **25.00** | **9.71** | **50.82** |
| **1698** | **19.90** | **17.93** | **22.03** |
| **1698** | **17.21** | **13.89** | **21.14** |

**LF estimates for Roman (2020) study are back calculated from overall LF rate primary & metastatic of 11% and p= 0.61**

**eFig 2(b)	Metastatic Lung Cancer BED ≥ 100Gy Single Fraction (any primary histology)**

| **N** | **2yr LR** | **LCI** | **UCI** |
| --- | --- | --- | --- |
| **25** | **17.00** | **6.73** | **36.77** |
| **129** | **13.75** | **8.81** | **20.83** |
| **45** | **32.70** | **20.68** | **47.53** |
| **199** | **20.42** | **15.15** | **26.95** |
| **199** | **21.05** | **9.98** | **39.06** |

**eFig 2(c)	Metastatic Lung Cancer BED < 100Gy Multi Fraction (any primary histology)**

| **N** | **2yr LR** | **LCI** | **UCI** |
| --- | --- | --- | --- |
| **84** | **16.50** | **10.00** | **26.01** |
| **18** | **19.40** | **6.96** | **43.64** |
| **20** | **42.60** | **23.42** | **64.29** |
| **44** | **9.20** | **3.52** | **21.98** |
| **49** | **17.00** | **8.86** | **30.15** |
| **65** | **69.10** | **56.92** | **79.10** |
| **149** | **46.90** | **39.03** | **54.92** |
| **429** | **38.74** | **33.71** | **44.02** |
| **429** | **29.24** | **15.61** | **47.99** |

**eFig 2(d)	Metastatic Lung Cancer BED < 100Gy Single Fraction (any primary histology)**

| **N** | **2yr LR** | **LCI** | **UCI** |
| --- | --- | --- | --- |
| **55** | **14.29** | **7.26** | **26.18** |
| **67** | **18.40** | **10.84** | **29.49** |
| **25** | **17.00** | **6.73** | **36.77** |
| **41** | **10.00** | **3.85** | **23.56** |
| **188** | **15.49** | **10.94** | **21.47** |
| **188** | **15.49** | **10.94** | **21.47** |

**eFig 3(a)	Metastatic Lung Cancer BED ≥ 100Gy Multi Fraction (colorectal primary)**

| **N** | **2yr LR** | **LCI** | **UCI** |
| --- | --- | --- | --- |
| **10** | **20.00** | **5.04** | **54.07** |
| **10** | **15.70** | **3.28** | **50.57** |
| **99** | **22.00** | **14.92** | **31.21** |
| **59** | **23.60** | **14.48** | **36.04** |
| **93** | **34.80** | **25.84** | **44.99** |
| **50** | **20.33** | **11.36** | **33.69** |
| 13 | **47.30** | **23.20** | **72.72** |
| 53 | **21.80** | **12.68** | **34.86** |
| 20 | **34.20** | **17.10** | **56.70** |
| **35** | **3.70** | **0.66** | **18.18** |
| **38** | **20.00** | **10.15** | **35.63** |
| **7** | **20.00** | **3.77** | **61.44** |
| **15** | **27.00** | **10.58** | **53.63** |
| **9** | **4.44** | **0.19** | **52.55** |
| **29** | **24.00** | **11.87** | **42.54** |
| **15** | **25.40** | **9.62** | **52.13** |
| **183** | **29.65** | **23.48** | **36.66** |
| **738** | **26.42** | **23.27** | **29.83** |
| **738** | **25.59** | **21.80** | **29.79** |

**eFig 3(b)	Metastatic Lung Cancer BED ≥ 100Gy Single Fraction (colorectal primary)**

| **N** | **2yr LR** | **LCI** | **UCI** |
| --- | --- | --- | --- |
| **41** | **25.02** | **14.13** | **40.35** |

**Osti 2018**

**eFig 3(c)	Metastatic Lung Cancer BED < 100Gy Single Fraction (colorectal primary)**

**Binkley 2015**

| **N** | **2yr LR** | | **LCI** | **UCI** |
| --- | --- | --- | --- | --- |
| **7** | **52.60** | **20.11** | | **83.03** |

**eFig 3(d)	Metastatic Lung Cancer BED < 100Gy Multi Fraction (colorectal primary)**

| **N** | **2yr LR** | **LCI** | **UCI** |
| --- | --- | --- | --- |
| **18** | **19.40** | **6.96** | **43.64** |
| **14** | **21.43** | **7.07** | **49.43** |
| **65** | **69.10** | **56.92** | **79.10** |
| **97** | **55.36** | **44.18** | **66.02** |
| **97** | **36.19** | **9.60** | **75.17** |

| Study | D1 | D2 | D3 | D4 | D5 | D6 | D7 | D8 | D9 |
| --- | --- | --- | --- | --- | --- | --- | --- | --- | --- |
| Alongi 2021 |  |  |  |  |  |  |  |  |  |
| Cummings 2018 |  |  |  |  |  |  |  |  |  |
| Huang 2023 |  |  |  |  |  |  |  |  |  |
| Jeppesen 2013 |  |  |  |  |  |  |  |  |  |
| Karasawa 2018 |  |  |  |  |  |  |  |  |  |
| Lee 2018 |  |  |  |  |  |  |  |  |  |
| Lucas 2014 |  |  |  |  |  |  |  |  |  |
| Ma 2018 |  |  |  |  |  |  |  |  |  |
| Mancini 2016 |  |  |  |  |  |  |  |  |  |
| Nittala 2021 |  |  |  |  |  |  |  |  |  |
| Scotti 2019 |  |  |  |  |  |  |  |  |  |
| Siva 2015 |  |  |  |  |  |  |  |  |  |
| Wang 2018 |  |  |  |  |  |  |  |  |  |

Judgement:

Star
 Unclear
 No Star

Table A1: Risk of bias summary plot for comparison studies

Table S1:

D1: Representativeness of exposed cohort
D2: Selection of non-exposed cohort
D3: Ascertainment of exposure
D4: Outcome not present at start of the study
D5: Comparability 1
D6: Comparability 2
D7: Assessment of outcome
D8: Length of follow up at least 2 years
D9: Adequacy of follow up

| **Study**  Tabe A2: Risk of bias summary plot for single-arm observational studies | **Representati-veness of exposed cohort** | **Ascertainment of exposure** | **Outcome not present at start of the study** | **Assessment of outcome** | **Length of follow up** | **Adequacy of follow up** |
| --- | --- | --- | --- | --- | --- | --- |
| Abdalmassih 2020 |  |  |  |  |  |  |
| Abe 2021 |  |  |  |  |  |  |
| Abreu 2018 |  |  |  |  |  |  |
| Ahmed 2018 |  |  |  |  |  |  |
| Aoki 2018 |  |  |  |  |  |  |
| Arnett 2019 |  |  |  |  |  |  |
| Baschnagel 2013 |  |  |  |  |  |  |
| Baumann 2009 |  |  |  |  |  |  |
| Berkovic 2020 |  |  |  |  |  |  |
| Bezjak 2019 |  |  |  |  |  |  |
| Bhandari 2016 |  |  |  |  |  |  |
| Binkley 2015 |  |  |  |  |  |  |
| Bral 2011 |  |  |  |  |  |  |
| Chai 2020 |  |  |  |  |  |  |
| Chang 2012 |  |  |  |  |  |  |
| Chang 2021 |  |  |  |  |  |  |
| Chaudhuri 2015 |  |  |  |  |  |  |
| Chiang 2016 |  |  |  |  |  |  |
| Choi 2023 |  |  |  |  |  |  |
| Dohopolski 2018 |  |  |  |  |  |  |
| Doupnik 2022 |  |  |  |  |  |  |
| Dubaere 2019 |  |  |  |  |  |  |
| Factor 2014 |  |  |  |  |  |  |
| Fakiris 2009 |  |  |  |  |  |  |
| Filippi 2014 |  |  |  |  |  |  |
| Fischer-Valuck 2015 |  |  |  |  |  |  |
| Franceschini 2017 |  |  |  |  |  |  |
| Fritz 2006 |  |  |  |  |  |  |
| Fritz 2008 |  |  |  |  |  |  |
| Haasbeek 2010 |  |  |  |  |  |  |
| Hamamoto 2009 |  |  |  |  |  |  |
| Hayashi 2022 |  |  |  |  |  |  |
| Heal 2015 |  |  |  |  |  |  |
| Helou 2017 |  |  |  |  |  |  |
| Hobbs 2017 |  |  |  |  |  |  |
| Hof 2003 |  |  |  |  |  |  |
| Hof 2007 |  |  |  |  |  |  |
| Hoyer 2006 |  |  |  |  |  |  |
| Lovoli 2023 |  |  |  |  |  |  |
| Jingu 2017 |  |  |  |  |  |  |
| Jung 2015 |  |  |  |  |  |  |
| Kalinauskaite 2020 |  |  |  |  |  |  |
| Kim 2009 |  |  |  |  |  |  |
| Kim 2013 |  |  |  |  |  |  |
| Kinj 2016 |  |  |  |  |  |  |
| Koba 2019 |  |  |  |  |  |  |
| Kobayashi 2020 |  |  |  |  |  |  |
| Koto 2007 |  |  |  |  |  |  |
| Kowalchuk 2021 |  |  |  |  |  |  |
| Kundu 2013 |  |  |  |  |  |  |
| Lagerwaard 2008 |  |  |  |  |  |  |
| Le 2006 |  |  |  |  |  |  |
| Lee 2018 |  |  |  |  |  |  |
| Lenglet 2019 |  |  |  |  |  |  |
| Li 2014 |  |  |  |  |  |  |
| Lischalk 2016(mets) |  |  |  |  |  |  |
| Lischalk 2016 |  |  |  |  |  |  |
| Lodeweges 2021 |  |  |  |  |  |  |
| Louie 2014 |  |  |  |  |  |  |
| Manyam 2019 |  |  |  |  |  |  |
| Matsuo 2011 |  |  |  |  |  |  |
| Meng 2019 |  |  |  |  |  |  |
| Mihai 2018 |  |  |  |  |  |  |
| Mix 2019 |  |  |  |  |  |  |
| Miyakawa 2012 |  |  |  |  |  |  |
| Miyakawa 2017 |  |  |  |  |  |  |
| Nagata 2005 |  |  |  |  |  |  |
| Nagata 2015 |  |  |  |  |  |  |
| Nath 2011 |  |  |  |  |  |  |
| Navarria 2014 |  |  |  |  |  |  |
| Nestle 2020 |  |  |  |  |  |  |
| Ng 2008 |  |  |  |  |  |  |
| Nicosia 2019 |  |  |  |  |  |  |
| Nicosia 2020 |  |  |  |  |  |  |
| Norihisa 2008 |  |  |  |  |  |  |
| Nyman 2016 |  |  |  |  |  |  |
| Oh 2012 |  |  |  |  |  |  |
| Okunieff 2006 |  |  |  |  |  |  |
| Olsen 2011 |  |  |  |  |  |  |
| Onimaru 2007 |  |  |  |  |  |  |
| Onishi 2011 |  |  |  |  |  |  |
| Osti 2018 |  |  |  |  |  |  |
| Patel 2020 |  |  |  |  |  |  |
| Qiu 2018 |  |  |  |  |  |  |
| Raman 2018 |  |  |  |  |  |  |
| Ricardi 2010 |  |  |  |  |  |  |
| Ricardi 2014 |  |  |  |  |  |  |
| Ricco 2017 |  |  |  |  |  |  |
| Roman 2020 |  |  |  |  |  |  |
| Rusthoven 2009 |  |  |  |  |  |  |
| Ryuno 2022 |  |  |  |  |  |  |
| S. Garcia-Cabezas 2015 |  |  |  |  |  |  |
| Schanne 2015 |  |  |  |  |  |  |
| Shintani 2017 |  |  |  |  |  |  |
| Shirata 2012 |  |  |  |  |  |  |
| Song 2009 |  |  |  |  |  |  |
| Song 2023 |  |  |  |  |  |  |
| Stephans 2018 |  |  |  |  |  |  |
| Sun 2017 |  |  |  |  |  |  |
| Takeda 2009 |  |  |  |  |  |  |
| Takeda 2011 |  |  |  |  |  |  |
| Takeda 2016 |  |  |  |  |  |  |
| Taremi 2012 |  |  |  |  |  |  |
| Testolin 2015 |  |  |  |  |  |  |
| Thibault 2014 |  |  |  |  |  |  |
| Timmerman 2010 |  |  |  |  |  |  |
| Timmerman 2018 |  |  |  |  |  |  |
| Trakul 2012 |  |  |  |  |  |  |
| Turzer 2011 |  |  |  |  |  |  |
| Van der Voort 2009 |  |  |  |  |  |  |
| Verstegen 2011 |  |  |  |  |  |  |
| Videtic 2021 |  |  |  |  |  |  |
| Watanabe 2021 |  |  |  |  |  |  |
| Wegner 2018 |  |  |  |  |  |  |
| Wulf 2004 |  |  |  |  |  |  |
| Xia 2006 |  |  |  |  |  |  |
| Yamashita 2014 |  |  |  |  |  |  |
| Yamamoto 2020 |  |  |  |  |  |  |
| Yang 2020 |  |  |  |  |  |  |
| Yaprak 2019 |  |  |  |  |  |  |
| Yoshitake 2015 |  |  |  |  |  |  |
| Zimmerman 2006 |  |  |  |  |  |  |

Table A3: Cochrane ROB2 tool for Randomised controlled trials

D1: Random sequence generation

Judgement:

Low risk

Some concerns

High risk

| RCTs | D1 | D2 | D3 | D4 | D5 | D6 | D7 |
| --- | --- | --- | --- | --- | --- | --- | --- |
| Singh 2019 |  |  |  |  |  |  |  |
| Siva 2021 |  |  |  |  |  |  |  |
| Videtic 2019 |  |  |  |  |  |  |  |

D2: Allocation concealment

D3: Blinding of participant and personnel

D4: Blinding of outcome assessment

D5: Incomplete outcome data

D6: Selective reporting (reporting bias)

D7: Overall risk of bias
